# Supplementary material for: A socioscientific issues approach to ninth-graders’ understanding of COVID-19 on health, wealth, and educational attainments
Source: PLoS One. 2023 Mar 27;18(3):e0280509. doi: 10.1371/journal.pone.0280509 (PMC10045461; doi:10.1371/journal.pone.0280509)
Supplement: S1 Fig — COVID-19 guidelines. (DOCX) [file pone.0280509.s001.docx]

What you should know about COVID-19 to protect yourself and others

#
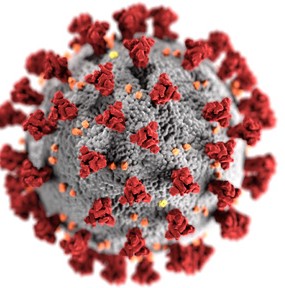
Know about COVID-19

- Coronavirus (COVID-19) is an illness caused by a virus that can spread from person

to person.

- The virus that causes COVID-19 is a new coronavirus that has spread throughout the world.
- COVID-19 symptoms can range from mild (or no symptoms) to severe illness.


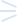

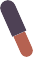

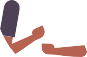

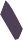

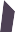

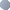


# Know how COVID-19 is spread

- You can become infected by coming into close contact (about 6 feet or two

arm lengths) with a person who has COVID-19. COVID-19 is primarily spread from person to person.

- You can become infected from respiratory droplets when an infected person coughs, sneezes, or talks.
- You may also be able to get it by touching a surface or object that has the virus on it, and then by touching your mouth, nose, or eyes.


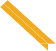

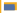

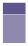


# Protect yourself and others from COVID-19

- There is currently no vaccine to protect against COVID-19. The best way to protect yourself is to avoid being exposed to the virus that causes COVID-19.
- Stay home as much as possible and avoid close contact with others.
- Wear a cloth face covering that covers your nose and mouth in public settings.
- Clean and disinfect frequently touched surfaces.
- Wash your hands often with soap and water for at least 20 seconds, or use an alcohol- based hand sanitizer that contains at least 60% alcohol.

# Practice social distancing

- Buy groceries and medicine, go to the doctor, and complete banking activities online when possible.
- If you must go in person, stay at least 6 feet away from


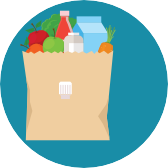

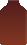


milk

others and disinfect items you must touch.

- Get deliveries and takeout, and limit in-person contact as much as possible.


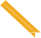

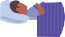

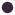


# Prevent the spread of COVID-19 if you are sick

- Stay home if you are sick, except to get medical care.
- Avoid public transportation, ride-sharing, or taxis.
- Separate yourself from other people and pets in your home.
- There is no specific treatment for COVID-19, but you can seek medical care to help relieve your symptoms.
-
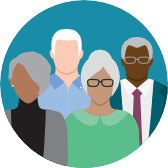
If you need medical attention, call ahead.

# Know your risk for severe illness

- Everyone is at risk of getting COVID-19.
- Older adults and people of any age who have serious underlying medical conditions may be at higher risk for more severe illness.


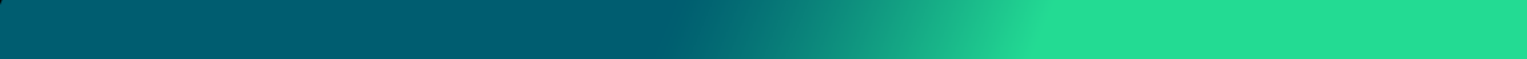


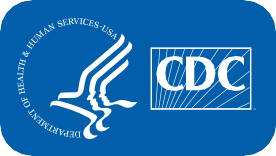
 [**cdc.gov/coronaviru**](http://www.cdc.gov/coronavirus)**s**

CS 314937A 04/15/2020
