# Supplementary figures and images for: A socioscientific issues approach to ninth-graders’ understanding of COVID-19 on health, wealth, and educational attainments
Source: PLoS One. 2023 Mar 27;18(3):e0280509. doi: 10.1371/journal.pone.0280509 (PMC10045461; doi:10.1371/journal.pone.0280509)

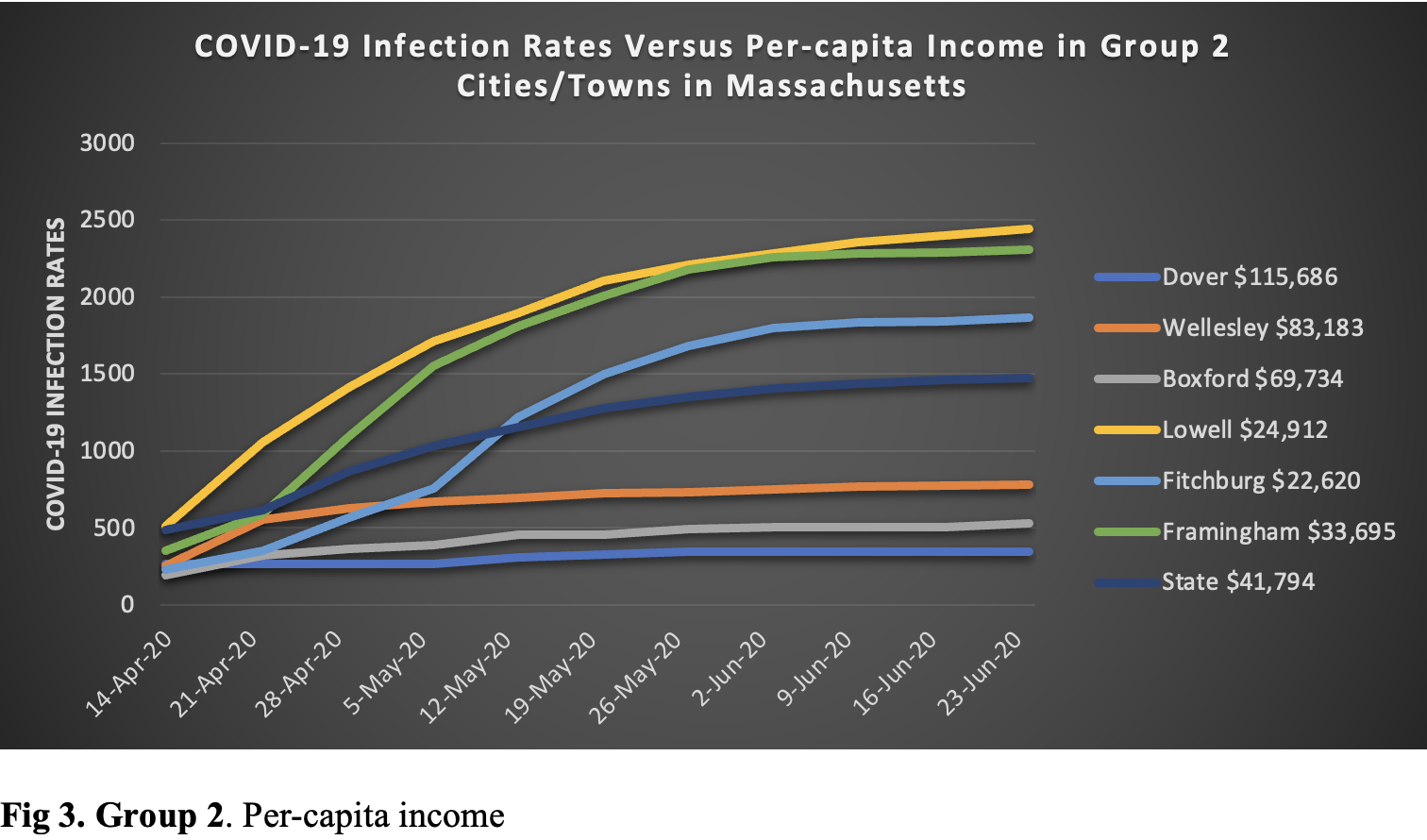

Supplement: S2 Fig — (TIF) [file pone.0280509.s002.tif]

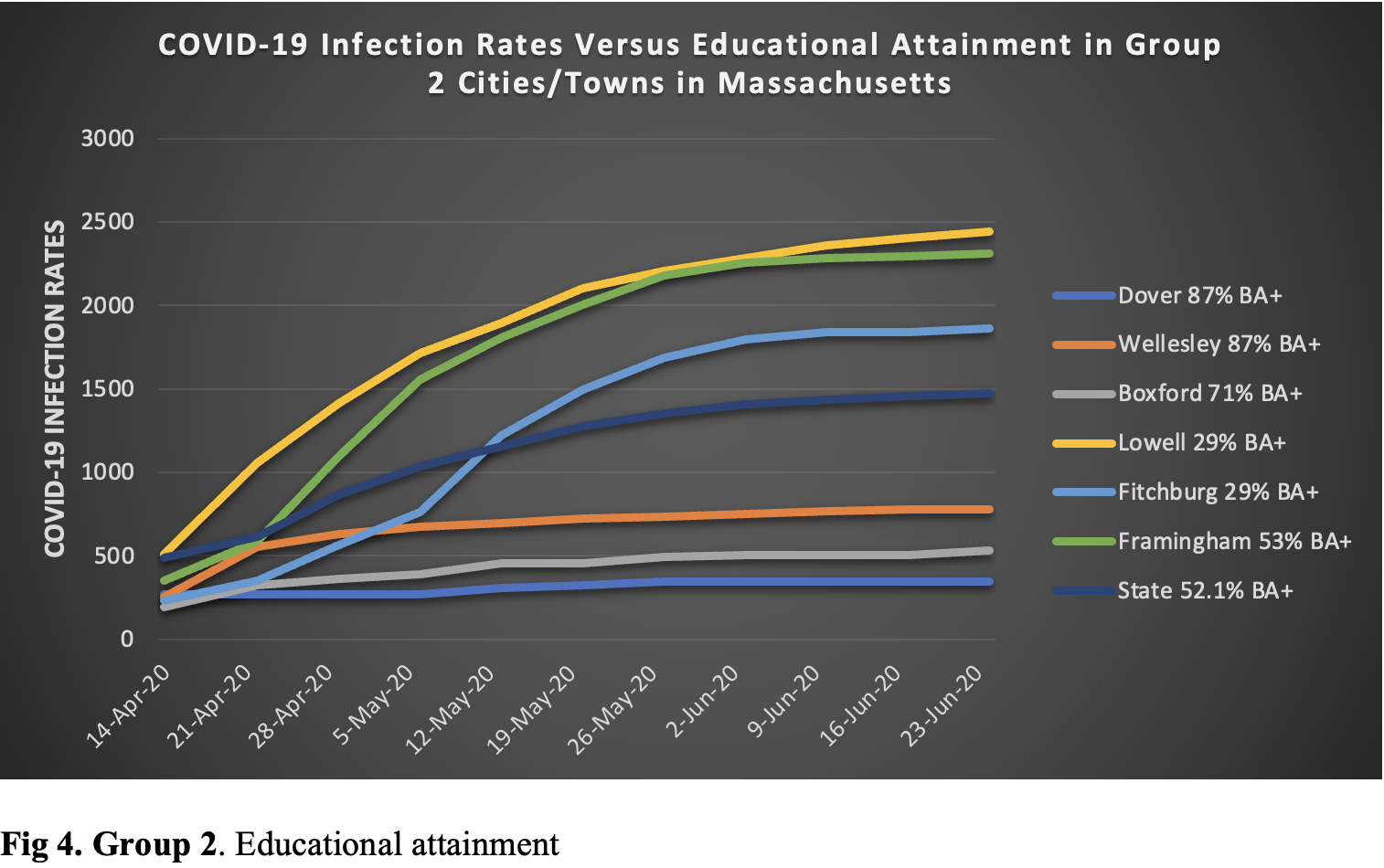

Supplement: S3 Fig — (TIF) [file pone.0280509.s003.tif]

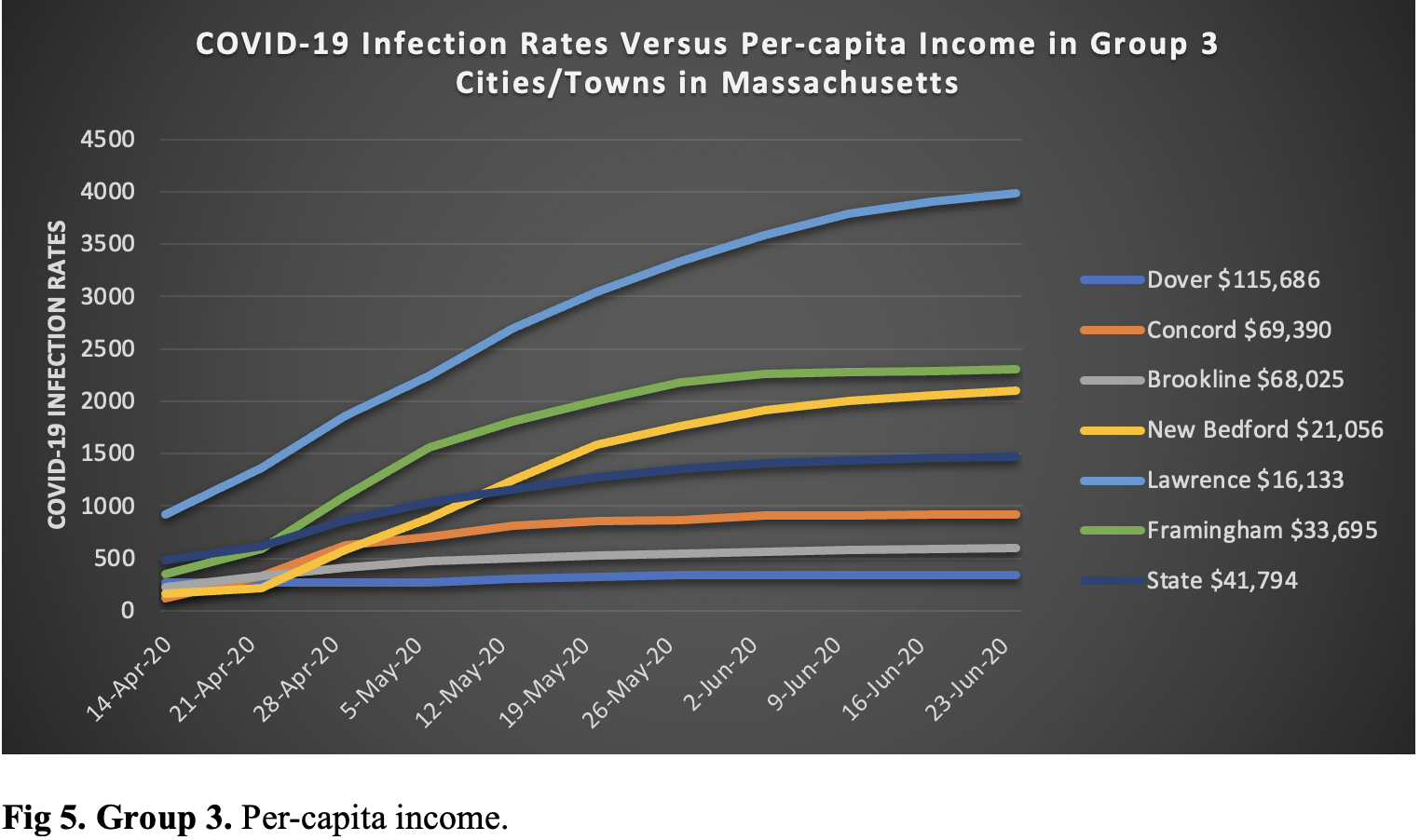

Supplement: S4 Fig — (TIF) [file pone.0280509.s004.tif]

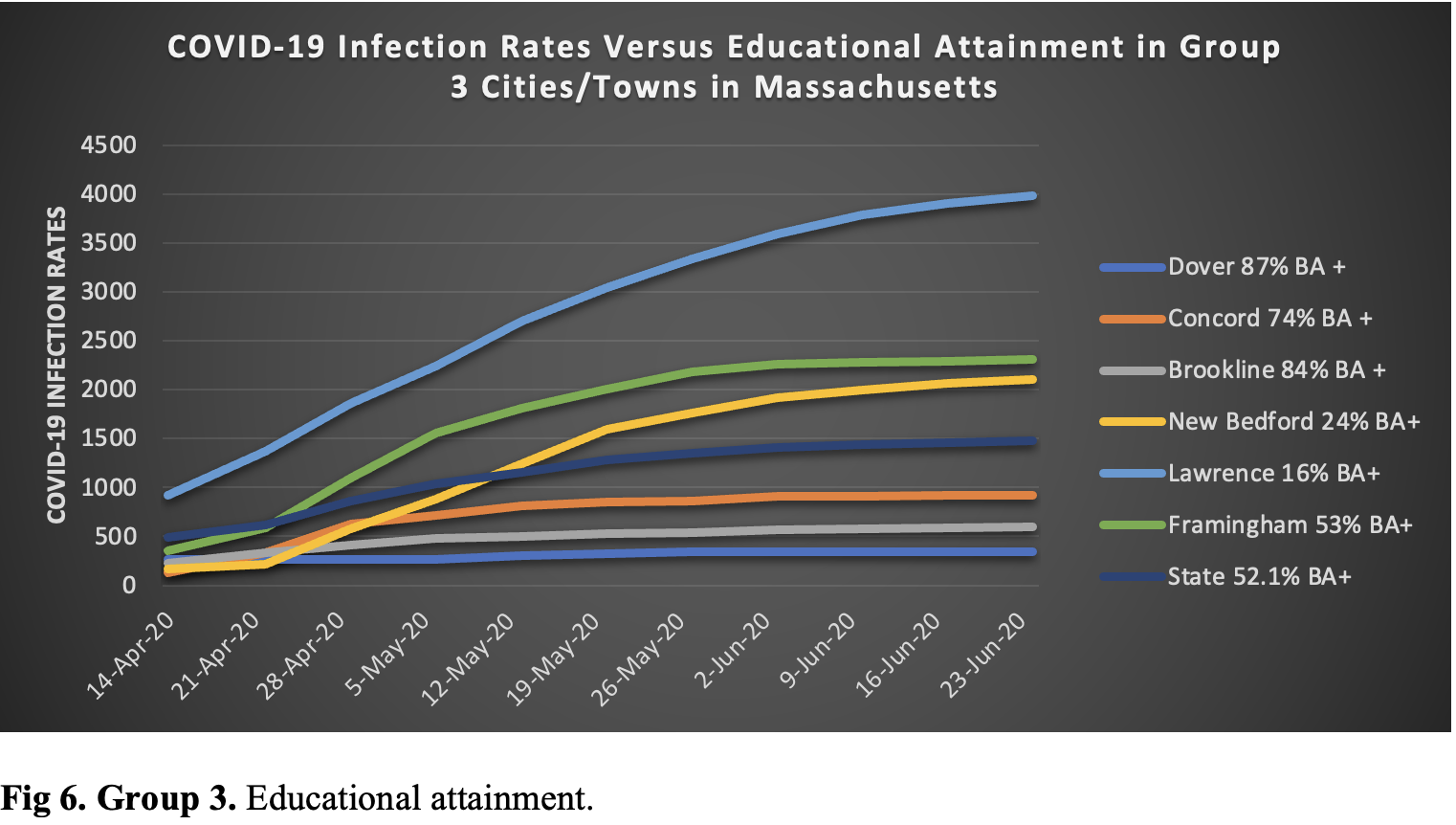

Supplement: S5 Fig — (TIF) [file pone.0280509.s005.tif]

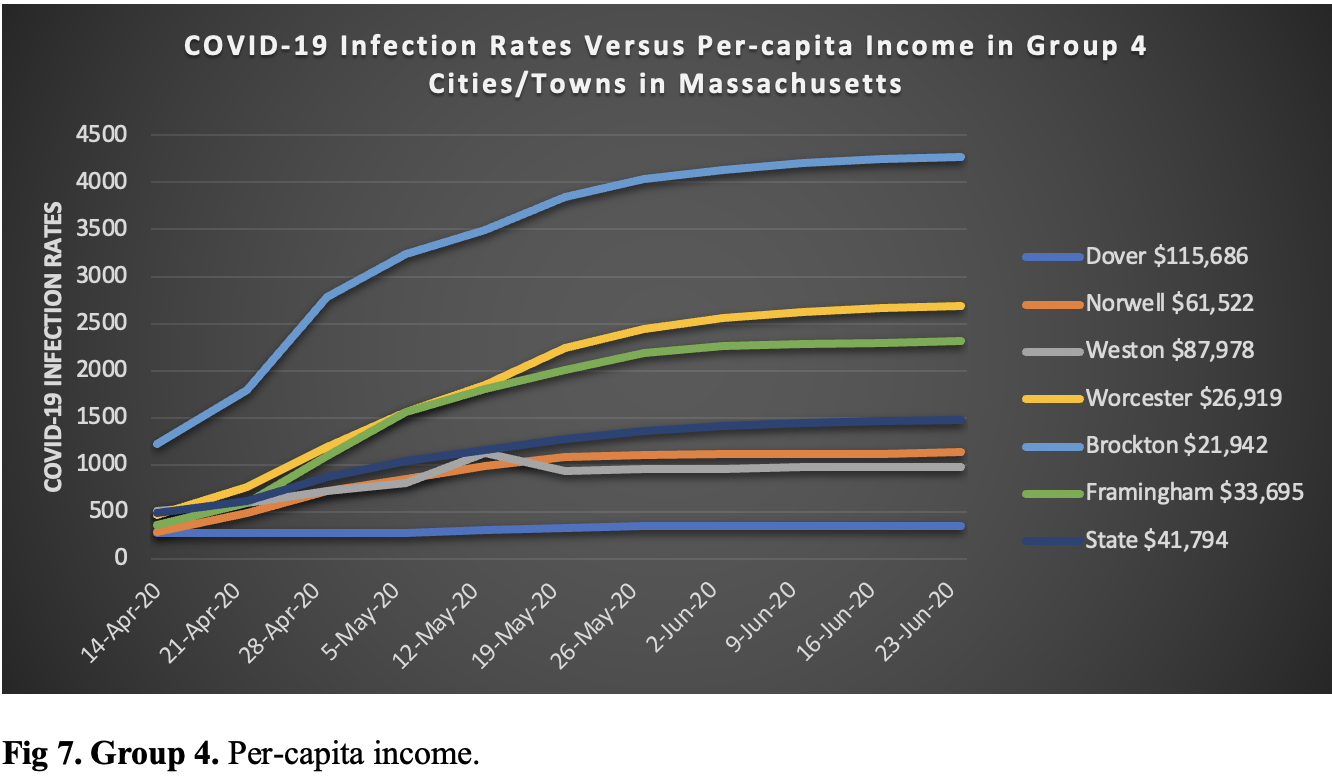

Supplement: S6 Fig — (TIF) [file pone.0280509.s006.tif]

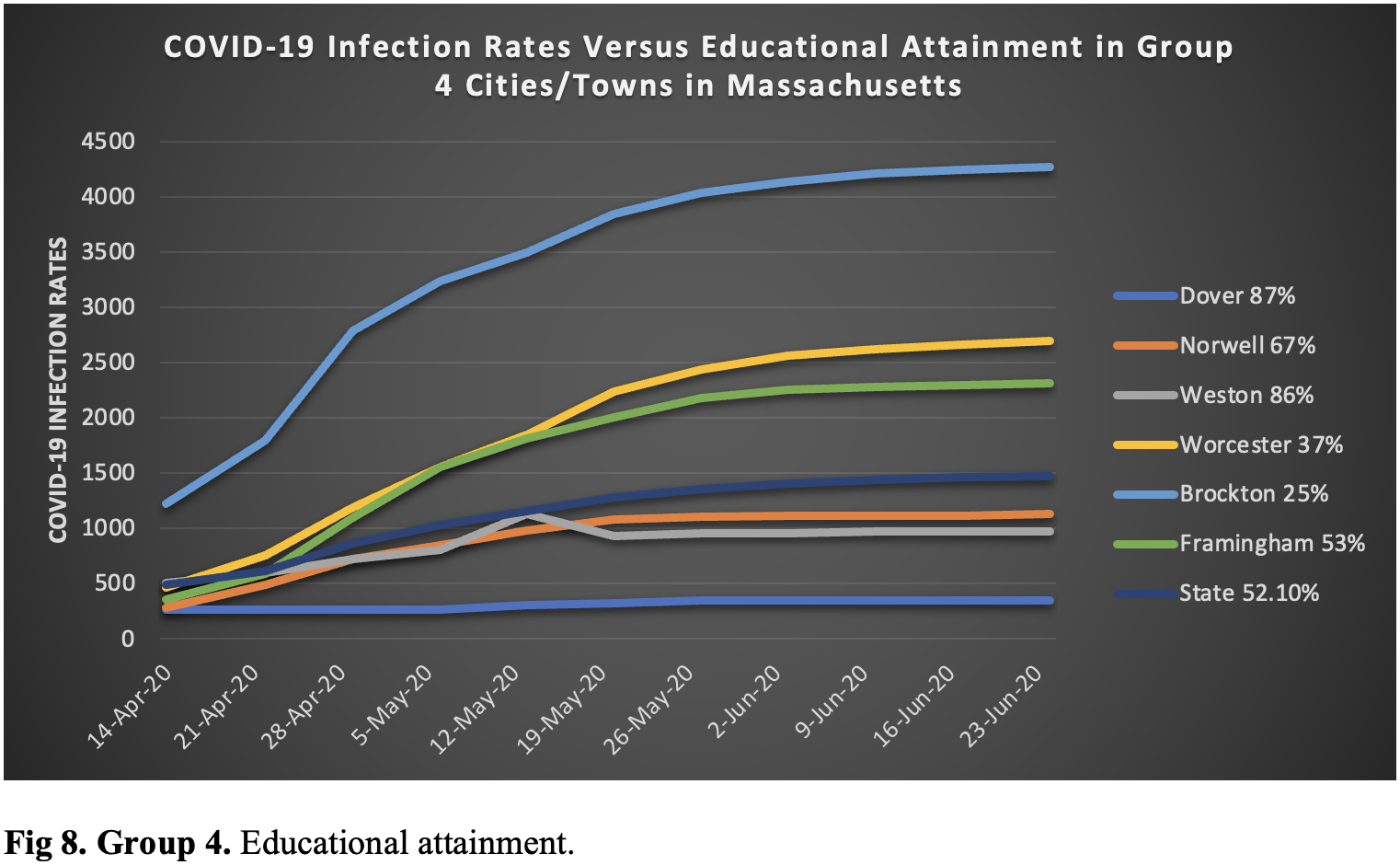

Supplement: S7 Fig — (TIF) [file pone.0280509.s007.tif]

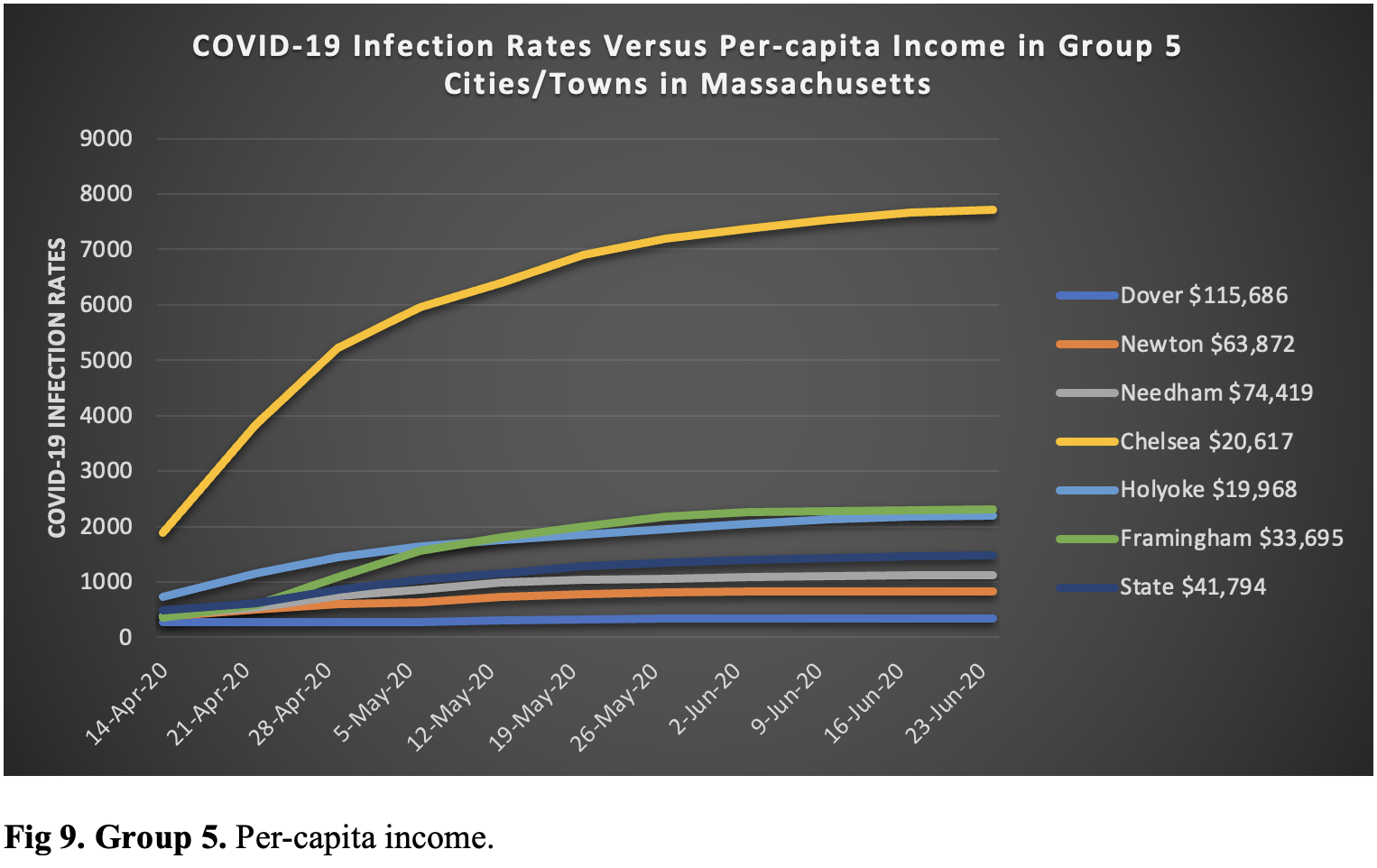

Supplement: S8 Fig — (TIF) [file pone.0280509.s008.tif]

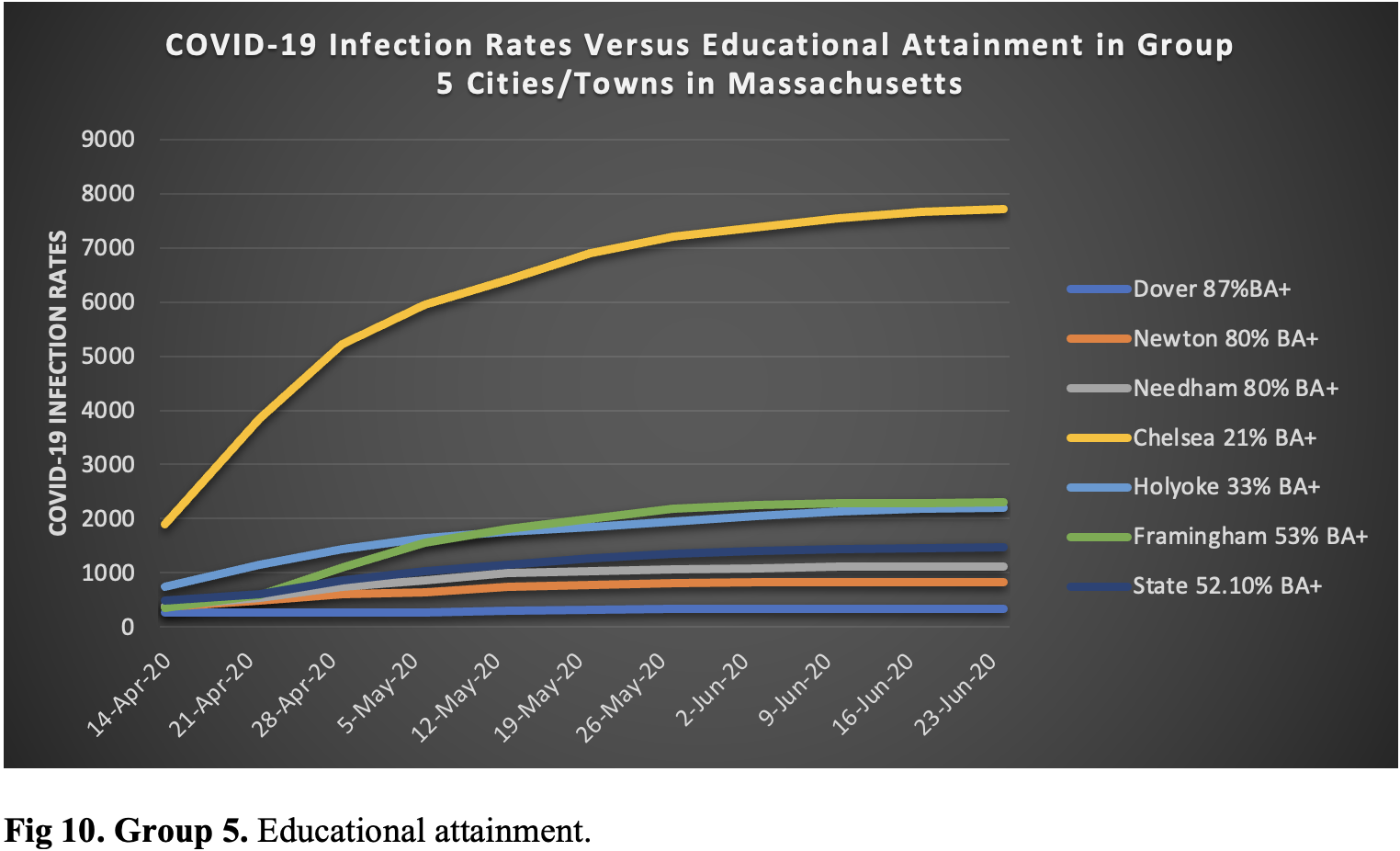

Supplement: S9 Fig — (TIF) [file pone.0280509.s009.tif]

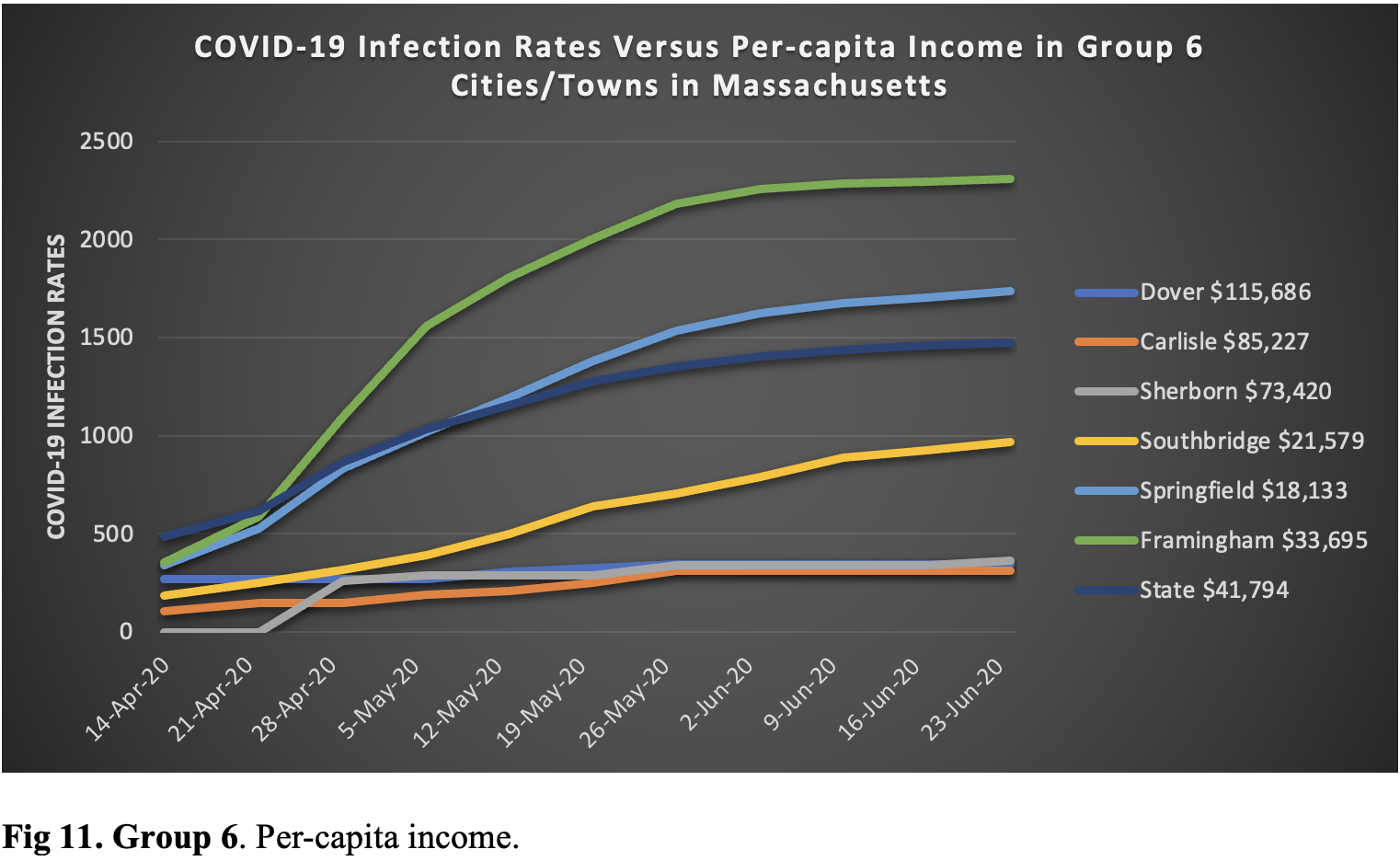

Supplement: S10 Fig — (TIF) [file pone.0280509.s010.tif]

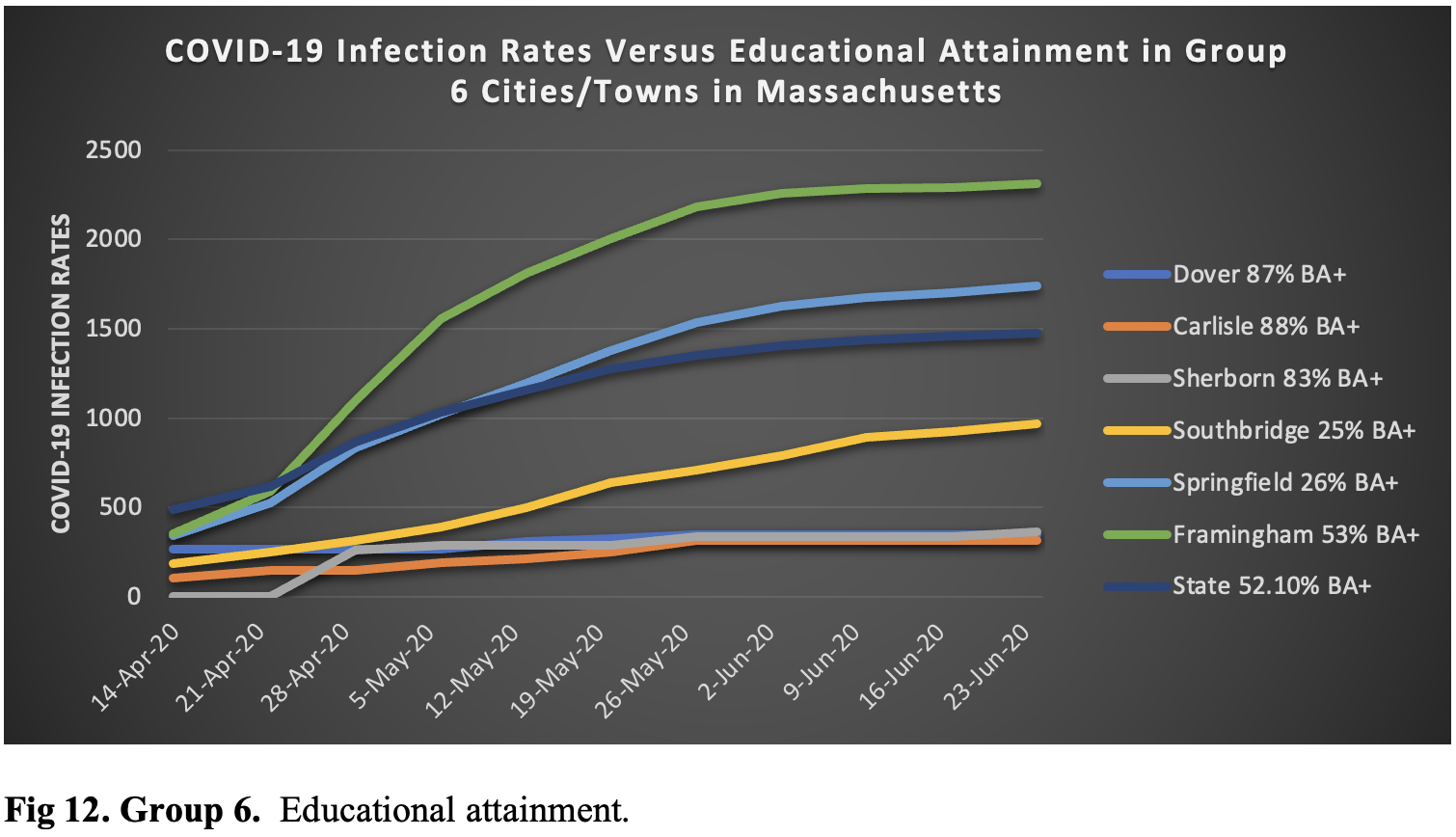

Supplement: S11 Fig — (TIF) [file pone.0280509.s011.tif]

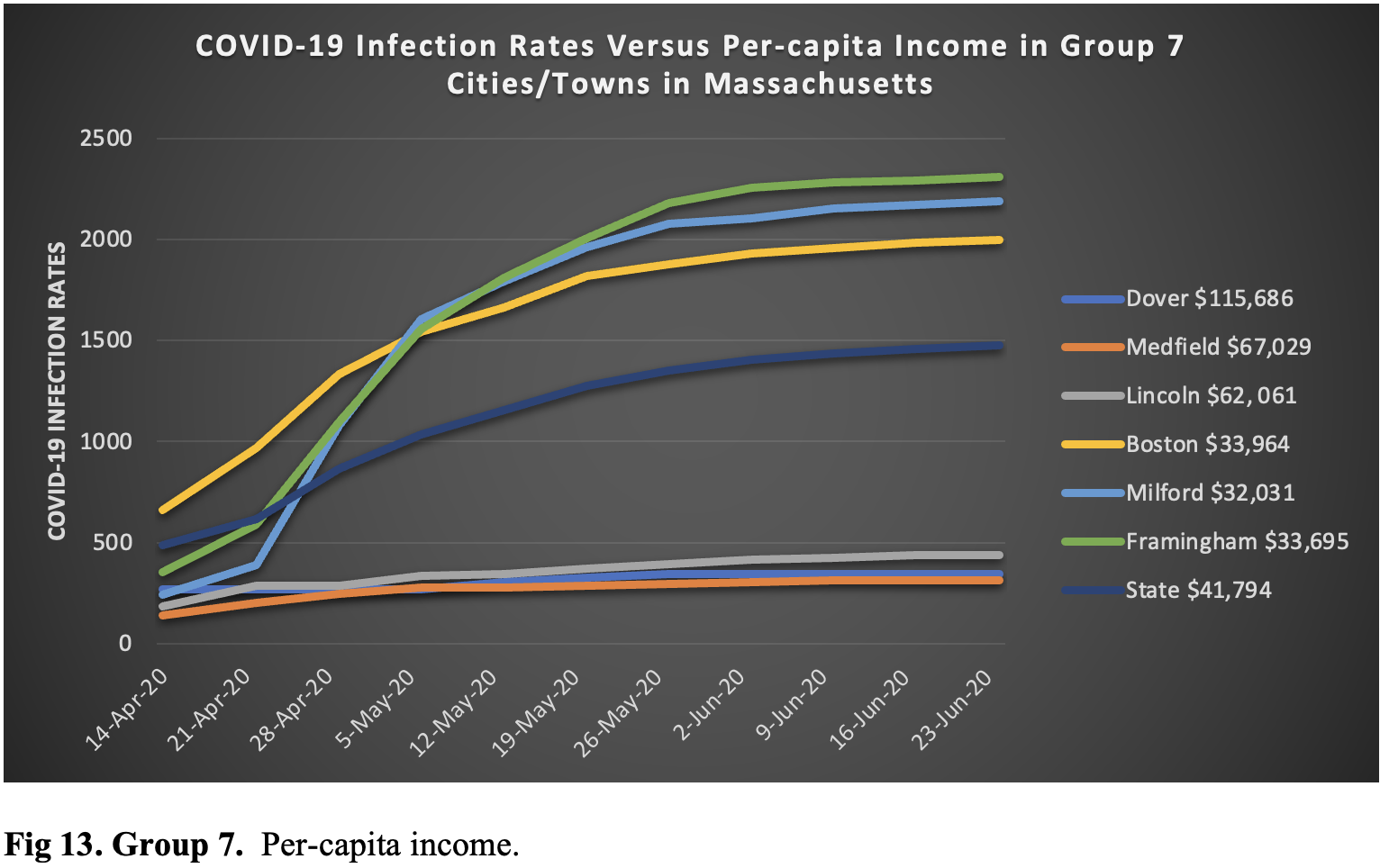

Supplement: S12 Fig — (TIF) [file pone.0280509.s012.tif]

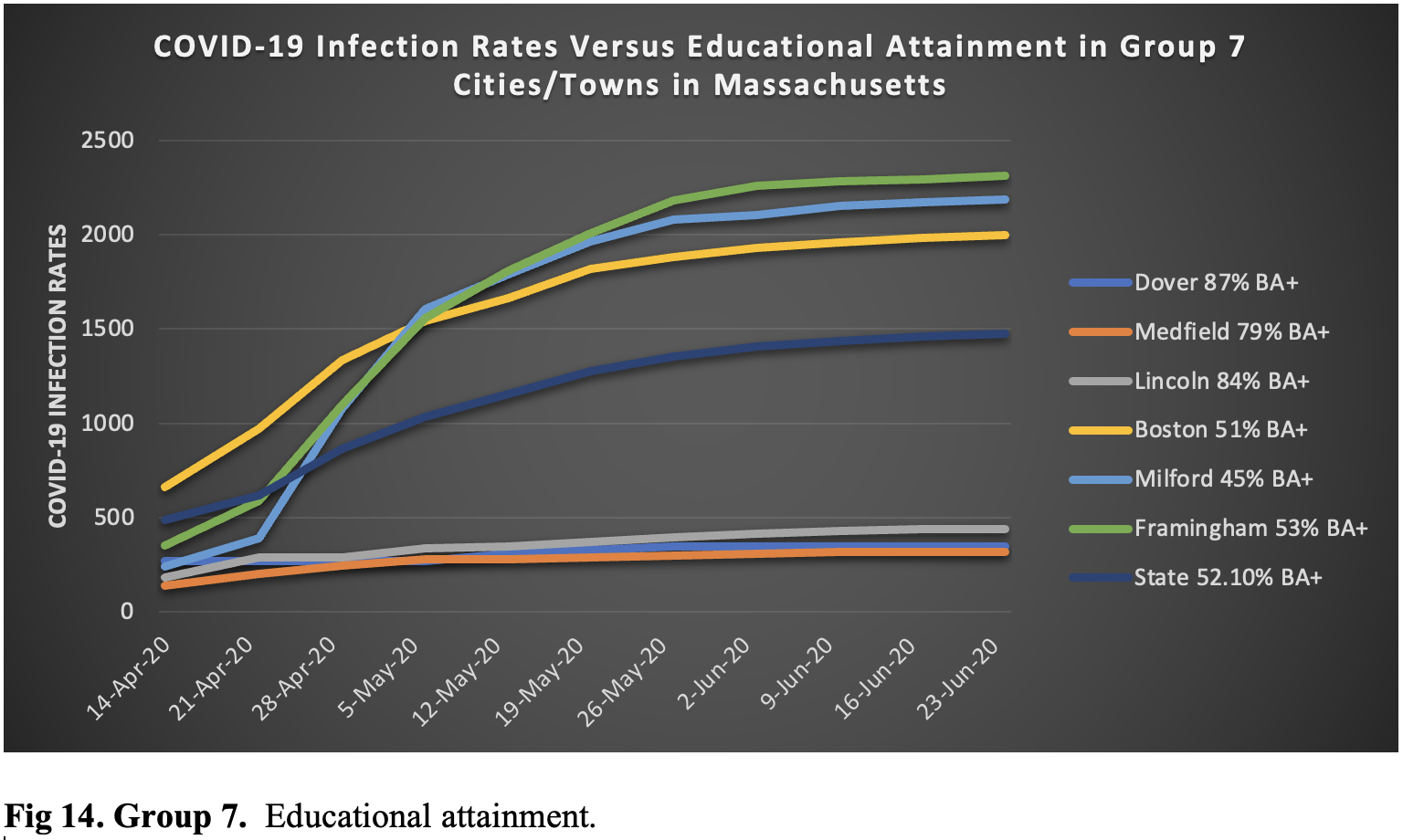

Supplement: S13 Fig — (TIF) [file pone.0280509.s013.tif]

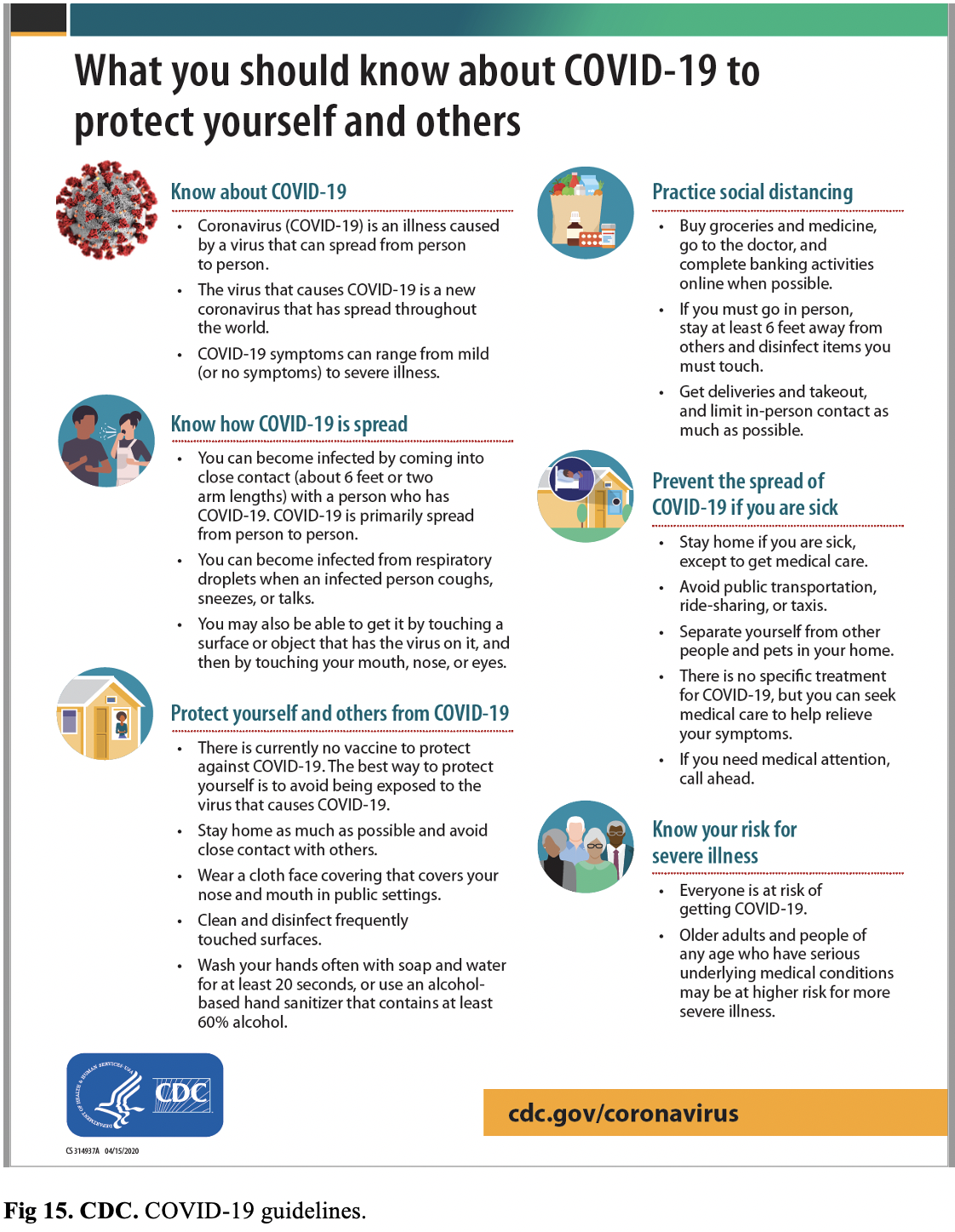

Supplement: S14 Fig — (TIF) [file pone.0280509.s014.tif]
