# Supplementary material for: A socioscientific issues approach to ninth-graders’ understanding of COVID-19 on health, wealth, and educational attainments
Source: PLoS One. 2023 Mar 27;18(3):e0280509. doi: 10.1371/journal.pone.0280509 (PMC10045461; doi:10.1371/journal.pone.0280509)
Supplement: S1 File — Table 7a. Group 2 per-capita incomes, educational attainment, and COVID-19 infection rates between January 1, 2020-April 29, 2020. Table 7b. Group 2 per-capita incomes, educational attainment, and COVID-19 infection rates between January 1, 2020-May 20, 2020. Table 7c. Group 2 per-capita incomes, educational attainment, and COVID-19 infection rates between January 1, 2020-June 10, 2020. Table 7d. Group 2 per-capita incomes, educational attainment, and COVID-19 infection rates between January 1, 2020-July 1, 2020. Table 8a. Group 3 per-capita incomes, educational attainment, and COVID-19 infection rates between January 1, 2020-April 29, 2020. Table 8b. Group 3 per-capita incomes, educational attainment, and COVID-19 infection rates between January 1, 2020-May 20, 2020. Table 8c. Group 3 per-capita incomes, educational attainment, and COVID-19 infection rates between January 1, 2020-June 10, 2020. Table 8d. Group 3 per-capita incomes, educational attainment, and COVID-19 infection rates between January 1, 2020-July 1, 2020. Table 9a. Group 4 per-capita incomes, educational attainment, and COVID-19 infection rates between January 1, 2020-April 29, 2020. Table 9b. Group 4 per-capita incomes, educational attainment, and COVID-19 infection rates between January 1, 2020-May 20, 2020. Table 9c. Group 4 per-capita incomes, educational attainment, and COVID-19 infection rates between January 1, 2020-June 10, 2020. Table 9d. Group 4 per-capita incomes, educational attainment, and COVID-19 infection rates between January 1, 2020-July 1, 2020. Table 10a. Group 5 per-capita incomes, educational attainment, and COVID-19 infection rates between January 1, 2020-April 29, 2020. Table 10b. Group 5 per-capita incomes, educational attainment, and COVID-19 infection rates between January 1, 2020-May 20, 2020. Table 10c Group 5 per-capita incomes, educational attainment, and COVID-19 infections rate between January 1, 2020-June 10, 2020. Table 10d. Group 5 per-capita incomes, educational [file pone.0280509.s015.docx]

S1 File.

**Table 7a. Group 2 per-capita incomes, educational attainment, and COVID-19 infection rates between January 1, 2020-April 29, 2020.**

| **Town** | **Per-capita Income** | **Educational Attainment in 2017** | | | | | **COVID-19**  **January 1-April 14, 2020** | | **COVID-19**  **January 1-April 22, 2020** | | **COVID-19**  **January 1-April 29, 2020** | |
| --- | --- | --- | --- | --- | --- | --- | --- | --- | --- | --- | --- | --- |
|  |  | **No HS Dip.** | **HS Dip/**  **GED** | **Some Coll.** | **AS or BA** | **Adv. Deg.** | **Count/ 100k** | **Rate/ 100k** | **Count/ 100k** | **Rate/ 100k** | **Count/ 100k** | **Rate/ 100k** |
| Dover | $115,686 | 2% | 5% | 6% | 40% | 47% | 14 | 268.65 | 14 | 268.65 | 14 | 268.65 |
| Wellesley | $83,183 | 2% | 6% | 6% | 37% | 50% | 75 | 252.23 | 164 | 551.54 | 187 | 628.90 |
| Boxford | $69,734 | 2% | 14% | 13% | 44% | 27% | 15 | 194.37 | 25 | 323.95 | 28 | 362.83 |
| Lowell | $24,912 | 20% | 34% | 17% | 20% | 9% | 598 | 511.73 | 1235 | 1056.83 | 1649 | 1411.11 |
| Fitchburg | $22,620 | 18% | 35% | 18% | 22% | 7% | 97 | 230.12 | 148 | 351.10 | 238 | 564.61 |
| Framingham | $33,695 | 11% | 23% | 13% | 33% | 20% | 263 | 353.18 | 438 | 588.19 | 816 | 1095.80 |
| **State** | **$41,794** | **9.3%** | **23.3%** | **15.3%** | **32%** | **20.10%** | **28,163** | **487.76** | **42,944** | **616.41** | **60,265** | **865.03** |

**Table 7b. Group 2 per-capita incomes, educational attainment, and COVID-19 infection rates between January 1, 2020-May 20, 2020.**

| **Town** | **Per-capita Income** | **Educational Attainment in 2017** | | | | | **COVID-19**  **January 1-May 6, 2020** | | **COVID-19**  **January 1-May 13, 2020** | | **COVID-19**  **January 1-May 20, 2020** | |
| --- | --- | --- | --- | --- | --- | --- | --- | --- | --- | --- | --- | --- |
|  |  | **No HS Dip.** | **HS Dip/**  **GED** | **Some Coll.** | **AS or BA** | **Adv. Deg.** | **Count/ 100k** | **Rate/ 100k** | **Count/ 100k** | **Rate/ 100k** | **Count/ 100k** | **Rate/ 100k** |
| Dover | $115,686 | 2% | 5% | 6% | 40% | 47% | 14 | 268.65 | 16 | 307.03 | 17 | 326.22 |
| Wellesley | $83,183 | 2% | 6% | 6% | 37% | 50% | 200 | 672.62 | 207 | 696.16 | 215 | 723.06 |
| Boxford | $69,734 | 2% | 14% | 13% | 44% | 27% | 30 | 388.74 | 35 | 453.53 | 35 | 453.53 |
| Lowell | $24,912 | 20% | 34% | 17% | 20% | 9% | 2002 | 1713.18 | 2215 | 1895.45 | 2457 | 2102.54 |
| Fitchburg | $22,620 | 18% | 35% | 18% | 22% | 7% | 320 | 759.15 | 516 | 1224.12 | 631 | 1496.94 |
| Framingham | $33,695 | 11% | 23% | 13% | 33% | 20% | 1159 | 1556.41 | 1347 | 1808.87 | 1493 | 2004.93 |
| **State** | **$41,794** | **9.3%** | **23.3%** | **15.3%** | **32%** | **20.10%** | **72025** | **1033.83** | **80,497** | **1155.44** | **88970** | **1277.06** |

**Table 7c. Group 2 per-capita incomes, educational attainment, and COVID-19 infection rates between January 1, 2020-June 10, 2020.**

| **Town** | **Per-capita Income** | **Educational Attainment in 2017** | | | | | **COVID-19**  **January 1-May 27, 2020** | | **COVID-19**  **January 1-June 3, 2020** | | **COVID-19**  **January 1-June 10, 2020** | |
| --- | --- | --- | --- | --- | --- | --- | --- | --- | --- | --- | --- | --- |
|  |  | **No HS Dip.** | **HS Dip/**  **GED** | **Some Coll.** | **AS or BA** | **Adv. Deg.** | **Count/ 100k** | **Rate/ 100k** | **Count/ 100k** | **Rate/ 100k** | **Count/ 100k** | **Rate/ 100k** |
| Dover | $115,686 | 2% | 5% | 6% | 40% | 47% | 18 | 345.41 | 18 | 345.41 | 18 | 345.41 |
| Wellesley | $83,183 | 2% | 6% | 6% | 37% | 50% | 218 | 733.15 | 223 | 749.97 | 228 | 766.78 |
| Boxford | $69,734 | 2% | 14% | 13% | 44% | 27% | 38 | 492.41 | 39 | 505.36 | 39 | 505.36 |
| Lowell | $24,912 | 20% | 34% | 17% | 20% | 9% | 2581 | 2208.65 | 2671 | 2285.67 | 2757 | 2359.26 |
| Fitchburg | $22,620 | 18% | 35% | 18% | 22% | 7% | 710 | 1684.35 | 758 | 1798.22 | 775 | 1838.55 |
| Framingham | $33,695 | 11% | 23% | 13% | 33% | 20% | 1625 | 2182.20 | 1681 | 2257.40 | 1700 | 2282.91 |
| **State** | **$41,794** | **9.3%** | **23.3%** | **15.3%** | **32%** | **20.10%** | **94220** | **1352.42** | **97964** | **1406.16** | **100158** | **1437.65** |

**Table 7d. Group 2 per-capita incomes, educational attainment, and COVID-19 infection rates between January 1, 2020-July 1, 2020.**

| **Town** | **Per-capita Income** | **Educational Attainment in 2017** | | | | | **COVID-19**  **January 1-June 17, 2020** | | **COVID-19**  **January 1-June 24, 2020** | | **COVID-19**  **January 1-July 1, 2020** | |
| --- | --- | --- | --- | --- | --- | --- | --- | --- | --- | --- | --- | --- |
|  |  | **No HS Dip.** | **HS Dip/**  **GED** | **Some Coll.** | **Count/100k** | **Rate/100k** | **Count/ 100k** | **Rate/ 100k** | **Count/ 100k** | **Rate/ 100k** | **Count/ 100k** | **Rate/ 100k** |
| Dover | $115,686 | 2% | 5% | 6% | 40% | 47% | 18 | 345.41 | 18 | 345.41 | 18 | 345.41 |
| Wellesley | $83,183 | 2% | 6% | 6% | 37% | 50% | 231 | 776.87 | 232 | 780.23 | 234 | 786.96 |
| Boxford | $69,734 | 2% | 14% | 13% | 44% | 27% | 39 | 505.36 | 41 | 531.28 | 42 | 544.24 |
| Lowell | $24,912 | 20% | 34% | 17% | 20% | 9% | 2807 | 2402.05 | 2854 | 2442.27 | 2895 | 2477.35 |
| Fitchburg | $22,620 | 18% | 35% | 18% | 22% | 7% | 776 | 1840.93 | 786 | 1864.65 | 795 | 1886.00 |
| Framingham | $33,695 | 11% | 23% | 13% | 33% | 20% | 1707 | 2292.31 | 1720 | 2309.77 | 1734 | 2328.57 |
| **State** | **$41,794** | **9.3%** | **23.3%** | **15.3%** | **32%** | **20.10%** | **101654** | **1459.12** | **102762** | **1475.03** | 103858 | 1490.76 |

**Table 8a. Group 3 per-capita incomes, educational attainment, and COVID-19 infection rates between January 1, 2020-April 29, 2020.**

| **Town** | **Per-capita Income** | **Educational Attainment in 2017** | | | | | **COVID-19**  **January 1-April 14, 2020** | | **COVID-19**  **January 1-April 22, 2020** | | **COVID-19**  **January 1-April 29, 2020** | |
| --- | --- | --- | --- | --- | --- | --- | --- | --- | --- | --- | --- | --- |
|  |  | **No HS Dip.** | **HS Dip/**  **GED** | **Some Coll.** | **AS or BA** | **Adv. Deg.** | **Count/ 100k** | **Rate/ 100k** | **Count/ 100k** | **Rate/ 100k** | **Count/ 100k** | **Rate/ 100k** |
| Dover | $115,686 | 2% | 5% | 6% | 40% | 47% | 14 | 268.65 | 14 | 268.65 | 14 | 268.65 |
| Concord | $69,390 | 6% | 12% | 8% | 34% | 40% | 23 | 122.83 | 63 | 336.45 | 117 | 624.83 |
| Brookline | $68,025 | 3% | 7% | 6% | 32% | 52% | 144 | 223.66 | 216 | 335.48 | 263 | 408.48 |
| New Bedford | $21,056 | 27% | 35% | 17% | 18% | 6% | 165 | 165.74 | 212 | 212.95 | 579 | 581.59 |
| Lawrence | $16,133 | 32% | 34% | 17% | 13% | 3% | 814 | 923.15 | 1205 | 1366.59 | 1639 | 1858.78 |
| Framingham | $33,695 | 11% | 23% | 13% | 33% | 20% | 263 | 353.18 | 438 | 588.19 | 816 | 1095.80 |
| **State** | **$41,794** | **9.3%** | **23.3%** | **15.3%** | **32%** | **20.10%** | **28,163** | **487.76** | **42,944** | **616.41** | **60,265** | **865.03** |

**Table 8b. Group 3 per-capita incomes, educational attainment, and COVID-19 infection rates between January 1, 2020-May 20, 2020.**

| **Town** | **Per-capita Income** | **Educational Attainment in 2017** | | | | | **COVID-19**  **January 1-May 6, 2020** | | **COVID-19**  **January 1-May 13, 2020** | | **COVID-19**  **January 1-May 20, 2020** | |
| --- | --- | --- | --- | --- | --- | --- | --- | --- | --- | --- | --- | --- |
|  |  | **No HS Dip.** | **HS Dip/**  **GED** | **Some Coll.** | **AS or BA** | **Adv. Deg.** | **Count/ 100k** | **Rate/ 100k** | **Count/ 100k** | **Rate/ 100k** | **Count/ 100k** | **Rate/ 100k** |
| Dover | $115,686 | 2% | 5% | 6% | 40% | 47% | 14 | 268.65 | 16 | 307.03 | 17 | 326.22 |
| Concord | $69,390 | 6% | 12% | 8% | 34% | 40% | 133 | 710.28 | 152 | 811.75 | 160 | 854.47 |
| Brookline | $68,025 | 3% | 7% | 6% | 32% | 52% | 308 | 478.38 | 321 | 498.57 | 343 | 532.74 |
| New Bedford | $21,056 | 27% | 35% | 17% | 18% | 6% | 881 | 884.94 | 1241 | 1246.55 | 1583 | 1590.07 |
| Lawrence | $16,133 | 32% | 34% | 17% | 13% | 3% | 1975 | 2239.84 | 2382 | 2701.42 | 2681 | 3040.51 |
| Framingham | $33,695 | 11% | 23% | 13% | 33% | 20% | 1159 | 1556.41 | 1347 | 1808.87 | 1493 | 2004.93 |
| **State** | **$41,794** | **9.3%** | **23.3%** | **15.3%** | **32%** | **20.10%** | **72025** | **1033.83** | **80,497** | **1155.44** | **88970** | **1277.06** |

**Table 8c. Group 3 per-capita incomes, educational attainment, and COVID-19 infection rates between January 1, 2020-June 10, 2020.**

| **Town** | **Per-capita Income** | **Educational Attainment in 2017** | | | | | **COVID-19**  **January 1-May 27, 2020** | | **COVID-19**  **January 1-June 3, 2020** | | **COVID-19**  **January 1-June 10, 2020** | |
| --- | --- | --- | --- | --- | --- | --- | --- | --- | --- | --- | --- | --- |
|  |  | **No HS Dip.** | **HS Dip/**  **GED** | **Some Coll.** | **AS or BA** | **Adv. Deg.** | **Count/ 100k** | **Rate/ 100k** | **Count/ 100k** | **Rate/ 100k** | **Count/ 100k** | **Rate/ 100k** |
| Dover | $115,686 | 2% | 5% | 6% | 40% | 47% | 18 | 345.41 | 18 | 345.41 | 18 | 345.41 |
| Concord | $69,390 | 6% | 12% | 8% | 34% | 40% | 162 | 865.16 | 170 | 907.88 | 170 | 907.88 |
| Brookline | $68,025 | 3% | 7% | 6% | 32% | 52% | 349 | 542.06 | 365 | 566.91 | 373 | 579.33 |
| New Bedford | $21,056 | 27% | 35% | 17% | 18% | 6% | 1756 | 1763.85 | 1910 | 1918.54 | 1991 | 1999.90 |
| Lawrence | $16,133 | 32% | 34% | 17% | 13% | 3% | 2939 | 3333.11 | 3163 | 3587.15 | 3339 | 3786.75 |
| Framingham | $33,695 | 11% | 23% | 13% | 33% | 20% | 1625 | 2182.20 | 1681 | 2257.40 | 1700 | 2282.91 |
| **State** | **$41,794** | **9.3%** | **23.3%** | **15.3%** | **32%** | **20.10%** | **94220** | **1352.42** | **97964** | **1406.16** | **100158** | **1437.65** |

**Table 8d. Group 3 per-capita incomes, educational attainment, and COVID-19 infection rates between January 1, 2020-July 1, 2020.**

| **Town** | **Per-capita Income** | **Educational Attainment in 2017** | | | | | **COVID-19**  **January 1-June 17, 2020** | | **COVID-19**  **January 1-June 24, 2020** | | **COVID-19**  **January 1-July 1, 2020** | |
| --- | --- | --- | --- | --- | --- | --- | --- | --- | --- | --- | --- | --- |
|  |  | **No HS Dip.** | **HS Dip/**  **GED** | **Some Coll.** | **AS or BA** | **Adv. Deg.** | **Count/ 100k** | **Rate/ 100k** | **Count/ 100k** | **Rate/ 100k** | **Count/ 100k** | **Rate/ 100k** |
| Dover | $115,686 | 2% | 5% | 6% | 40% | 47% | 18 | 345.41 | 18 | 345.41 | 18 | 345.41 |
| Concord | $69,390 | 6% | 12% | 8% | 34% | 40% | 172 | 918.56 | 173 | 923.90 | 170 | 907.88 |
| Brookline | $68,025 | 3% | 7% | 6% | 32% | 52% | 379 | 588.65 | 386 | 599.52 | 393 | 610.40 |
| New Bedford | $21,056 | 27% | 35% | 17% | 18% | 6% | 2052 | 2061.17 | 2095 | 2104.36 | 2138 | 2147.55 |
| Lawrence | $16,133 | 32% | 34% | 17% | 13% | 3% | 3443 | 3904.69 | 3515 | 3986.35 | 3553 | 4029.45 |
| Framingham | $33,695 | 11% | 23% | 13% | 33% | 20% | 1707 | 2292.31 | 1720 | 2309.77 | 1734 | 2328.57 |
| **State** | **$41,794** | **9.3%** | **23.3%** | **15.3%** | **32%** | **20.10%** | **101654** | **1459.12** | **102762** | **1475.03** | 103858 | 1490.76 |

**Table 9a. Group 4 per-capita incomes, educational attainment, and COVID-19 infection rates between January 1, 2020-April 29, 2020.**

| **Town** | **Per-capita Income** | **Educational Attainment in 2017** | | | | | **COVID-19**  **January 1-April 14, 2020** | | **COVID-19**  **January 1-April 22, 2020** | | **COVID-19**  **January 1-April 29, 2020** | |
| --- | --- | --- | --- | --- | --- | --- | --- | --- | --- | --- | --- | --- |
|  |  | **No HS Dip.** | **HS Dip/**  **GED** | **Some Coll.** | **AS or BA** | **Adv. Deg.** | **Count/ 100k** | **Rate/ 100k** | **Count/ 100k** | **Rate/ 100k** | **Count/ 100k** | **Rate/ 100k** |
| Dover | $115,686 | 2% | 5% | 6% | 40% | 47% | 14 | 268.65 | 14 | 268.65 | 14 | 268.65 |
| Norwell | $61,522 | 3% | 18% | 12% | 43% | 24% | 30 | 280.25 | 52 | 485.76 | 77 | 719.30 |
| Weston | $87,978 | 1% | 7% | 6% | 34% | 52% | 56 | 504.79 | 66 | 594.93 | 80 | 721.13 |
| Worcester | $26,919 | 16% | 30% | 17% | 26% | 11% | 886 | 461.77 | 1457 | 759.37 | 2284 | 1190.40 |
| Brockton | $21,942 | 19% | 35% | 21% | 20% | 5% | 1202 | 1223.43 | 1763 | 1794.43 | 2735 | 2783.76 |
| Framingham | $33,695 | 11% | 23% | 13% | 33% | 20% | 263 | 353.18 | 438 | 588.19 | 816 | 1095.80 |
| **State** | **$41,794** | **9.3%** | **23.3%** | **15.3%** | **32%** | **20.10%** | **28,163** | **487.76** | **42,944** | **616.41** | **60,265** | **865.03** |

**Table 9b. Group 4 per-capita incomes, educational attainment, and COVID-19 infection rates between January 1, 2020-May 20, 2020.**

| **Town** | **Per-capita Income** | **Educational Attainment in 2017** | | | | | **COVID-19**  **January 1-May 6, 2020** | | **COVID-19**  **January 1-May 13, 2020** | | **COVID-19**  **January 1-May 20, 2020** | |
| --- | --- | --- | --- | --- | --- | --- | --- | --- | --- | --- | --- | --- |
|  |  | **No HS Dip.** | **HS Dip/**  **GED** | **Some Coll.** | **AS or BA** | **Adv. Deg.** | **Count/ 100k** | **Rate/ 100k** | **Count/ 100k** | **Rate/ 100k** | **Count/ 100k** | **Rate/ 100k** |
| Dover | $115,686 | 2% | 5% | 6% | 40% | 47% | 14 | 268.65 | 16 | 307.03 | 17 | 326.22 |
| Norwell | $61,522 | 3% | 18% | 12% | 43% | 24% | 91 | 850.08 | 105 | 980.86 | 116 | 1083.62 |
| Weston | $87,978 | 1% | 7% | 6% | 34% | 52% | 89 | 802.25 | 125 | 1126.76 | 103 | 928.45 |
| Worcester | $26,919 | 16% | 30% | 17% | 26% | 11% | 2989 | 1557.84 | 3549 | 1849.70 | 4299 | 2240.60 |
| Brockton | $21,942 | 19% | 35% | 21% | 20% | 5% | 3179 | 3235.68 | 3429 | 3490.14 | 3773 | 3840.27 |
| Framingham | $33,695 | 11% | 23% | 13% | 33% | 20% | 1159 | 1556.41 | 1347 | 1808.87 | 1493 | 2004.93 |
| **State** | **$41,794** | **9.3%** | **23.3%** | **15.3%** | **32%** | **20.10%** | **72025** | **1033.83** | **80,497** | **1155.44** | **88970** | **1277.06** |

**Table 9c. Group 4 per-capita incomes, educational attainment, and COVID-19 infection rates between January 1, 2020-June 10, 2020.**

| **Town** | **Per-capita Income** | **Educational Attainment in 2017** | | | | | **COVID-19**  **January 1-May 27, 2020** | | **COVID-19**  **January 1-June 3, 2020** | | **COVID-19**  **January 1-June 10, 2020** | |
| --- | --- | --- | --- | --- | --- | --- | --- | --- | --- | --- | --- | --- |
|  |  | **No HS Dip.** | **HS Dip/**  **GED** | **Some Coll.** | **AS or BA** | **Adv. Deg.** | **Count/ 100k** | **Rate/ 100k** | **Count/ 100k** | **Rate/ 100k** | **Count/ 100k** | **Rate/ 100k** |
| Dover | $115,686 | 2% | 5% | 6% | 40% | 47% | 18 | 345.41 | 18 | 345.41 | 18 | 345.41 |
| Norwell | $61,522 | 3% | 18% | 12% | 43% | 24% | 118 | 1102.30 | 119 | 1111.64 | 119 | 1111.64 |
| Weston | $87,978 | 1% | 7% | 6% | 34% | 52% | 106 | 955.49 | 106 | 955.49 | 108 | 973.52 |
| Worcester | $26,919 | 16% | 30% | 17% | 26% | 11% | 4681 | 2439.69 | 4910 | 2559.04 | 5028 | 2620.54 |
| Brockton | $21,942 | 19% | 35% | 21% | 20% | 5% | 3961 | 4031.62 | 4064 | 4136.46 | 4136 | 4209.74 |
| Framingham | $33,695 | 11% | 23% | 13% | 33% | 20% | 1625 | 2182.20 | 1681 | 2257.40 | 1700 | 2282.91 |
| **State** | **$41,794** | **9.3%** | **23.3%** | **15.3%** | **32%** | **20.10%** | **94220** | **1352.42** | **97964** | **1406.16** | **100158** | **1437.65** |

**Table 9d. Group 4 per-capita incomes, educational attainment, and COVID-19 infection rates between January 1, 2020-July 1, 2020.**

| **Town** | **Per-capita Income** | **Educational Attainment in 2017** | | | | | **COVID-19**  **January 1-June 17, 2020** | | **COVID-19**  **January 1-June 24, 2020** | | **COVID-19**  **January 1-July 1, 2020** | |
| --- | --- | --- | --- | --- | --- | --- | --- | --- | --- | --- | --- | --- |
|  |  | **No HS Dip.** | **HS Dip/**  **GED** | **Some Coll.** | **AS or BA** | **Adv. Deg.** | **Count/ 100k** | **Rate/ 100k** | **Count/ 100k** | **Rate/ 100k** | **Count/ 100k** | **Rate/ 100k** |
| Dover | $115,686 | 2% | 5% | 6% | 40% | 47% | 18 | 345.41 | 18 | 345.41 | 18 | 345.41 |
| Norwell | $61,522 | 3% | 18% | 12% | 43% | 24% | 119 | 1111.64 | 121 | 1130.32 | 122 | 1139.67 |
| Weston | $87,978 | 1% | 7% | 6% | 34% | 52% | 108 | 973.52 | 108 | 973.52 | 108 | 973.52 |
| Worcester | $26,919 | 16% | 30% | 17% | 26% | 11% | 5112 | 2664.32 | 5165 | 2691.95 | 5227 | 2724.25 |
| Brockton | $21,942 | 19% | 35% | 21% | 20% | 5% | 4172 | 4246.39 | 4197 | 4271.83 | 4225 | 4300.33 |
| Framingham | $33,695 | 11% | 23% | 13% | 33% | 20% | 1707 | 2292.31 | 1720 | 2309.77 | 1734 | 2328.57 |
| **State** | **$41,794** | **9.3%** | **23.3%** | **15.3%** | **32%** | **20.10%** | **101654** | **1459.12** | **102762** | **1475.03** | 103858 | 1490.76 |

**Table 10a. Group 5 per-capita incomes, educational attainment, and COVID-19 infection rates between January 1, 2020-April 29, 2020.**

| **Town** | **Per-capita Income** | **Educational Attainment in 2017** | | | | | **COVID-19**  **January 1-April 14, 2020** | | **COVID-19**  **January 1-April 22, 2020** | | **COVID-19**  **January 1-April 29, 2020** | |
| --- | --- | --- | --- | --- | --- | --- | --- | --- | --- | --- | --- | --- |
|  |  | **No HS Dip.** | **HS Dip/**  **GED** | **Some Coll.** | **AS or BA** | **Adv. Deg.** | **Count/ 100k** | **Rate/ 100k** | **Count/ 100k** | **Rate/ 100k** | **Count/ 100k** | **Rate/ 100k** |
| Dover | $115,686 | 2% | 5% | 6% | 40% | 47% | 14 | 268.65 | 14 | 268.65 | 14 | 268.65 |
| Newton | $63,872 | 3% | 9% | 7% | 32% | 48% | 337 | 367.39 | 455 | 496.03 | 553 | 602.87 |
| Needham | $74,419 | 2% | 10% | 8% | 38% | 42% | 114 | 388.92 | 156 | 532.20 | 217 | 740.31 |
| Chelsea | $20,617 | 33% | 33% | 13% | 14% | 7% | 712 | 1890.37 | 1447 | 3841.80 | 1965 | 5217.10 |
| Holyoke | $19,968 | 21% | 28% | 19% | 23% | 10% | 305 | 740.21 | 474 | 1150.36 | 596 | 1446.45 |
| Framingham | $33,695 | 11% | 23% | 13% | 33% | 20% | 263 | 353.18 | 438 | 588.19 | 816 | 1095.80 |
| **State** | **$41,794** | **9.3%** | **23.3%** | **15.3%** | **32%** | **20.10%** | **28,163** | **487.76** | **42,944** | **616.41** | **60,265** | **865.03** |

**Table 10b. Group 5 per-capita incomes, educational attainment, and COVID-19 infection rates between January 1, 2020-May 20, 2020.**

| **Town** | **Per-capita Income** | **Educational Attainment in 2017** | | | | | **COVID-19**  **January 1-May 6, 2020** | | **COVID-19**  **January 1-May 13, 2020** | | **COVID-19**  **January 1-May 20, 2020** | |
| --- | --- | --- | --- | --- | --- | --- | --- | --- | --- | --- | --- | --- |
|  |  | **No HS Dip.** | **HS Dip/**  **GED** | **Some Coll.** | **AS or BA** | **Adv. Deg.** | **Count/ 100k** | **Rate/ 100k** | **Count/ 100k** | **Rate/ 100k** | **Count/ 100k** | **Rate/ 100k** |
| Dover | $115,686 | 2% | 5% | 6% | 40% | 47% | 14 | 268.65 | 16 | 307.03 | 17 | 326.22 |
| Newton | $63,872 | 3% | 9% | 7% | 32% | 48% | 588 | 641.03 | 675 | 735.87 | 710 | 774.03 |
| Needham | $74,419 | 2% | 10% | 8% | 38% | 42% | 251 | 856.30 | 292 | 996.17 | 303 | 1033.70 |
| Chelsea | $20,617 | 33% | 33% | 13% | 14% | 7% | 2244 | 5957.85 | 2412 | 6403.89 | 2598 | 6897.72 |
| Holyoke | $19,968 | 21% | 28% | 19% | 23% | 10% | 678 | 1645.46 | 723 | 1754.67 | 764 | 1854.17 |
| Framingham | $33,695 | 11% | 23% | 13% | 33% | 20% | 1159 | 1556.41 | 1347 | 1808.87 | 1493 | 2004.93 |
| **State** | **$41,794** | **9.3%** | **23.3%** | **15.3%** | **32%** | **20.10%** | **72025** | **1033.83** | **80,497** | **1155.44** | **88970** | **1277.06** |

**Table 10c Group 5 per-capita incomes, educational attainment, and COVID-19 infections rate between January 1, 2020-June 10, 2020.**

| **Town** | **Per-capita Income** | **Educational Attainment in 2017** | | | | | **COVID-19**  **January 1-May 27, 2020** | | **COVID-19**  **January 1-June 3, 2020** | | **COVID-19**  **January 1-June 10, 2020** | |
| --- | --- | --- | --- | --- | --- | --- | --- | --- | --- | --- | --- | --- |
|  |  | **No HS Dip.** | **HS Dip/**  **GED** | **Some Coll.** | **AS or BA** | **Adv. Deg.** | **Count/ 100k** | **Rate/ 100k** | **Count/ 100k** | **Rate/ 100k** | **Count/ 100k** | **Rate/ 100k** |
| Dover | $115,686 | 2% | 5% | 6% | 40% | 47% | 18 | 345.41 | 18 | 345.41 | 18 | 345.41 |
| Newton | $63,872 | 3% | 9% | 7% | 32% | 48% | 745 | 812.19 | 760 | 828.54 | 767 | 836.17 |
| Needham | $74,419 | 2% | 10% | 8% | 38% | 42% | 311 | 1060.99 | 318 | 1084.87 | 325 | 1108.75 |
| Chelsea | $20,617 | 33% | 33% | 13% | 14% | 7% | 2713 | 7203.05 | 2779 | 7378.28 | 2839 | 7537.58 |
| Holyoke | $19,968 | 21% | 28% | 19% | 23% | 10% | 804 | 1951.25 | 842 | 2043.47 | 877 | 2128.41 |
| Framingham | $33,695 | 11% | 23% | 13% | 33% | 20% | 1625 | 2182.20 | 1681 | 2257.40 | 1700 | 2282.91 |
| **State** | **$41,794** | **9.3%** | **23.3%** | **15.3%** | **32%** | **20.10%** | **94220** | **1352.42** | **97964** | **1406.16** | **100158** | **1437.65** |

**Table 10d. Group 5 per-capita incomes, educational attainment, and COVID-19 infection rates between January 1, 2020-July 1, 2020.**

| **Town** | **Per-capita Income** | **Educational Attainment in 2017** | | | | | **COVID-19**  **January 1-June 17, 2020** | | **COVID-19**  **January 1-June 24, 2020** | | **COVID-19**  **January 1-July 1, 2020** | |
| --- | --- | --- | --- | --- | --- | --- | --- | --- | --- | --- | --- | --- |
|  |  | **No HS Dip.** | **HS Dip/**  **GED** | **Some Coll.** | **AS or BA** | **Adv. Deg.** | **Count/ 100k** | **Rate/ 100k** | **Count/ 100k** | **Rate/ 100k** | **Count/ 100k** | **Rate/ 100k** |
| Dover | $115,686 | 2% | 5% | 6% | 40% | 47% | 18 | 345.41 | 18 | 345.41 | 18 | 345.41 |
| Newton | $63,872 | 3% | 9% | 7% | 32% | 48% | 765 | 833.99 | 767 | 836.17 | 773 | 842.71 |
| Needham | $74,419 | 2% | 10% | 8% | 38% | 42% | 328 | 1118.99 | 329 | 1122.40 | 330 | 1125.81 |
| Chelsea | $20,617 | 33% | 33% | 13% | 14% | 7% | 2885 | 7659.63 | 2907 | 7718.12 | 2944 | 7816.28 |
| Holyoke | $19,968 | 21% | 28% | 19% | 23% | 10% | 899 | 2181.81 | 905 | 2196.37 | 913 | 2215.78 |
| Framingham | $33,695 | 11% | 23% | 13% | 33% | 20% | 1707 | 2292.31 | 1720 | 2309.77 | 1734 | 2328.57 |
| **State** | **$41,794** | **9.3%** | **23.3%** | **15.3%** | **32%** | **20.10%** | **101654** | **1459.12** | **102762** | **1475.03** | 103858 | 1490.76 |

**Table 11a. Group 6 per-capita incomes, educational attainment, and COVID-19 infection rates between January 1, 2020-April 29, 2020.**

| **Town** | **Per-capita Income** | **Educational Attainment in 2017** | | | | | **COVID-19**  **January 1-April 14, 2020** | | **COVID-19**  **January 1-April 22, 2020** | | **COVID-19**  **January 1-April 29, 2020** | |
| --- | --- | --- | --- | --- | --- | --- | --- | --- | --- | --- | --- | --- |
|  |  | **No HS Dip.** | **HS Dip/**  **GED** | **Some Coll.** | **AS or BA** | **Adv. Deg.** | **Count/ 100k** | **Rate/ 100k** | **Count/ 100k** | **Rate/ 100k** | **Count/ 100k** | **Rate/ 100k** |
| Dover | $115,686 | 2% | 5% | 6% | 40% | 47% | 14 | 268.65 | 14 | 268.65 | 14 | 268.65 |
| Carlisle | $85,227 | 2% | 5% | 5% | 41% | 47% | 5 | 104.85 | 7 | 146.79 | 7 | 146.79 |
| Sherborn | $73,420 | 1% | 9% | 6% | 38% | 45% | <5 | * | 5 | * | 10 | 260.60 |
| Southbridge | $21,579 | 17% | 35% | 22% | 19% | 6% | 31 | 184.05 | 42 | 249.36 | 53 | 314.67 |
| Springfield | $18,133 | 23% | 31% | 20% | 19% | 7% | 542 | 342.32 | 835 | 527.37 | 1317 | 831.79 |
| Framingham | $33,695 | 11% | 23% | 13% | 33% | 20% | 263 | 353.18 | 438 | 588.19 | 816 | 1095.80 |
| **State** | **$41,794** | **9.3%** | **23.3%** | **15.3%** | **32%** | **20.10%** | **28,163** | **487.76** | **42,944** | **616.41** | **60,265** | **865.03** |

**Table 11b. Group 6 per-capita incomes, educational attainment, and COVID-19 infection rates between January 1, 2020-May 20, 2020.**

| **Town** | **Per-capita Income** | **Educational Attainment in 2017** | | | | | **COVID-19**  **January 1-May 6, 2020** | | **COVID-19**  **January 1-May 13, 2020** | | **COVID-19**  **January 1-May 20, 2020** | |
| --- | --- | --- | --- | --- | --- | --- | --- | --- | --- | --- | --- | --- |
|  |  | **No HS Dip.** | **HS Dip/**  **GED** | **Some Coll.** | **AS or BA** | **Adv. Deg.** | **Count/ 100k** | **Rate/ 100k** | **Count/ 100k** | **Rate/ 100k** | **Count/ 100k** | **Rate/ 100k** |
| Dover | $115,686 | 2% | 5% | 6% | 40% | 47% | 14 | 268.65 | 16 | 307.03 | 17 | 326.22 |
| Carlisle | $85,227 | 2% | 5% | 5% | 41% | 47% | 9 | 188.73 | 10 | 209.70 | 12 | 251.64 |
| Sherborn | $73,420 | 1% | 9% | 6% | 38% | 45% | 11 | 286.66 | 11 | 286.66 | 11 | 286.66 |
| Southbridge | $21,579 | 17% | 35% | 22% | 19% | 6% | 66 | 391.85 | 84 | 498.72 | 108 | 641.21 |
| Springfield | $18,133 | 23% | 31% | 20% | 19% | 7% | 1615 | 1020.00 | 1889 | 1193.05 | 2186 | 1380.63 |
| Framingham | $33,695 | 11% | 23% | 13% | 33% | 20% | 1159 | 1556.41 | 1347 | 1808.87 | 1493 | 2004.93 |
| **State** | **$41,794** | **9.3%** | **23.3%** | **15.3%** | **32%** | **20.10%** | **72025** | **1033.83** | **80,497** | **1155.44** | **88970** | **1277.06** |

**Table 11c. Group 6 per-capita incomes, educational attainment, and COVID-19 infection rates between January 1, 2020-June 10, 2020.**

| **Town** | **Per-capita Income** | **Educational Attainment in 2017** | | | | | **COVID-19**  **January 1-May 27, 2020** | | **COVID-19**  **January 1-June 3, 2020** | | **COVID-19**  **January 1-June 10, 2020** | |
| --- | --- | --- | --- | --- | --- | --- | --- | --- | --- | --- | --- | --- |
|  |  | **No HS Dip.** | **HS Dip/**  **GED** | **Some Coll.** | **AS or BA** | **Adv. Deg.** | **Count/ 100k** | **Rate/ 100k** | **Count/ 100k** | **Rate/ 100k** | **Count/ 100k** | **Rate/ 100k** |
| Dover | $115,686 | 2% | 5% | 6% | 40% | 47% | 18 | 345.41 | 18 | 345.41 | 18 | 345.41 |
| Carlisle | $85,227 | 2% | 5% | 5% | 41% | 47% | 15 | 314.55 | 15 | 314.55 | 15 | 314.55 |
| Sherborn | $73,420 | 1% | 9% | 6% | 38% | 45% | 13 | 338.78 | 13 | 338.78 | 13 | 338.78 |
| Southbridge | $21,579 | 17% | 35% | 22% | 19% | 6% | 119 | 706.51 | 133 | 789.63 | 150 | 890.56 |
| Springfield | $18,133 | 23% | 31% | 20% | 19% | 7% | 2432 | 1536.00 | 2573 | 1625.06 | 2650 | 1673.69 |
| Framingham | $33,695 | 11% | 23% | 13% | 33% | 20% | 1625 | 2182.20 | 1681 | 2257.40 | 1700 | 2282.91 |
| **State** | **$41,794** | **9.3%** | **23.3%** | **15.3%** | **32%** | **20.10%** | **94220** | **1352.42** | 97964 | 1406.16 | **100158** | **1437.65** |

**Table 11d. Group 6 per-capita incomes, educational attainment, and COVID-19 infection rates between January 1, 2020-July 1, 2020.**

| **Town** | **Per-capita Income** | **Educational Attainment in 2017** | | | | | **COVID-19**  **January 1-June 17, 2020** | | **COVID-19**  **January 1-June 24, 2020** | | **COVID-19**  **January 1-July 1, 2020** | |
| --- | --- | --- | --- | --- | --- | --- | --- | --- | --- | --- | --- | --- |
|  |  | **No HS Dip.** | **HS Dip/**  **GED** | **Some Coll.** | **AS or BA** | **Adv. Deg.** | **Count/ 100k** | **Rate/ 100k** | **Count/ 100k** | **Rate/ 100k** | **Count/ 100k** | **Rate/ 100k** |
| Dover | $115,686 | 2% | 5% | 6% | 40% | 47% | 18 | 345.41 | 18 | 345.41 | 18 | 345.41 |
| Carlisle | $85,227 | 2% | 5% | 5% | 41% | 47% | 15 | 314.55 | 15 | 314.55 | 17 | 356.49 |
| Sherborn | $73,420 | 1% | 9% | 6% | 38% | 45% | 13 | 338.78 | 14 | 364.84 | 15 | 390.90 |
| Southbridge | $21,579 | 17% | 35% | 22% | 19% | 6% | 156 | 926.19 | 163 | 967.75 | 168 | 997.43 |
| Springfield | $18,133 | 23% | 31% | 20% | 19% | 7% | 2697 | 1703.37 | 2754 | 1739.37 | 2833 | 1789.27 |
| Framingham | $33,695 | 11% | 23% | 13% | 33% | 20% | 1707 | 2292.31 | 1720 | 2309.77 | 1734 | 2328.57 |
| **State** | **$41,794** | **9.3%** | **23.3%** | **15.3%** | **32%** | **20.10%** | **101654** | **1459.12** | **102762** | **1475.03** | 103858 | 1490.76 |

**Table 12a. Group 7 per-capita incomes, educational attainment, and COVID-19 infection rates between January 1, 2020-April 29, 2020.**

| **Town** | **Per-capita Income** | **Educational Attainment in 2017** | | | | | **COVID-19**  **January 1-April 14, 2020** | | **COVID-19**  **January 1-April 22, 2020** | | **COVID-19**  **January 1-April 29, 2020** | |
| --- | --- | --- | --- | --- | --- | --- | --- | --- | --- | --- | --- | --- |
|  |  | **No HS Dip.** | **HS Dip/**  **GED** | **Some Coll.** | **AS or BA** | **Adv. Deg.** | **Count/ 100k** | **Rate/ 100k** | **Count/ 100k** | **Rate/ 100k** | **Count/ 100k** | **Rate/ 100k** |
| Dover | $115,686 | 2% | 5% | 6% | 40% | 47% | 14 | 268.65 | 14 | 268.65 | 14 | 268.65 |
| Medfield | $67,029 | 1% | 9% | 11% | 42% | 37% | 16 | 140.35 | 23 | 201.75 | 28 | 245.61 |
| Lincoln | $62, 061 | 1% | 4% | 10% | 36% | 48% | 16 | 184.91 | 25 | 288.92 | 25 | 288.92 |
| Boston | $33,964 | 14% | 21% | 13% | 30% | 21% | 4609 | 663.20 | 6744 | 970.40 | 9284 | 1335.89 |
| Milford | $32,031 | 8% | 27% | 20% | 33% | 12% | 71 | 242.29 | 114 | 389.04 | 316 | 1078.38 |
| Framingham | $33,695 | 11% | 23% | 13% | 33% | 20% | 263 | 353.18 | 438 | 588.19 | 816 | 1095.80 |
| **State** | **$41,794** | **9.3%** | **23.3%** | **15.3%** | **32%** | **20.10%** | **28,163** | **487.76** | **42,944** | **616.41** | **60,265** | **865.03** |

**Table 12b. Group 7 per-capita incomes, educational attainment, and COVID-19 infection rates between January 1, 2020-May 20, 2020.**

| **Town** | **Per-capita Income** | **Educational Attainment in 2017** | | | | | **COVID-19**  **January 1-May 6, 2020** | | **COVID-19**  **January 1-May 13, 2020** | | **COVID-19**  **January 1-May 20, 2020** | |
| --- | --- | --- | --- | --- | --- | --- | --- | --- | --- | --- | --- | --- |
|  |  | **No HS Dip.** | **HS Dip/**  **GED** | **Some Coll.** | **AS or BA** | **Adv. Deg.** | **Count/ 100k** | **Rate/ 100k** | **Count/ 100k** | **Rate/ 100k** | **Count/ 100k** | **Rate/ 100k** |
| Dover | $115,686 | 2% | 5% | 6% | 40% | 47% | 14 | 268.65 | 16 | 307.03 | 17 | 326.22 |
| Medfield | $67,029 | 1% | 9% | 11% | 42% | 37% | 32 | 280.69 | 32 | 280.69 | 33 | 289.46 |
| Lincoln | $62, 061 | 1% | 4% | 10% | 36% | 48% | 29 | 335.15 | 30 | 346.70 | 32 | 369.82 |
| Boston | $33,964 | 14% | 21% | 13% | 30% | 21% | 10729 | 1543.81 | 11551 | 1662.09 | 12629 | 1817.20 |
| Milford | $32,031 | 8% | 27% | 20% | 33% | 12% | 470 | 1603.92 | 525 | 1791.61 | 575 | 1962.24 |
| Framingham | $33,695 | 11% | 23% | 13% | 33% | 20% | 1159 | 1556.41 | 1347 | 1808.87 | 1493 | 2004.93 |
| **State** | **$41,794** | **9.3%** | **23.3%** | **15.3%** | **32%** | **20.10%** | **72025** | **1033.83** | **80,497** | **1155.44** | **88970** | **1277.06** |

**Table 12c. Group 7 per-capita incomes, educational attainment, and COVID-19 infection rates between January 1, 2020-June 10, 2020.**

| **Town** | **Per-capita Income** | **Educational Attainment in 2017** | | | | | **COVID-19**  **January 1-May 27, 2020** | | **COVID-19**  **January 1-June 3, 2020** | | **COVID-19**  **January 1-June 10, 2020** | |
| --- | --- | --- | --- | --- | --- | --- | --- | --- | --- | --- | --- | --- |
|  |  | **No HS Dip.** | **HS Dip/**  **GED** | **Some Coll.** | **AS or BA** | **Adv. Deg.** | **Count/ 100k** | **Rate/ 100k** | **Count/ 100k** | **Rate/ 100k** | **Count/ 100k** | **Rate/ 100k** |
| Dover | $115,686 | 2% | 5% | 6% | 40% | 47% | 18 | 345.41 | 18 | 345.41 | 18 | 345.41 |
| Medfield | $67,029 | 1% | 9% | 11% | 42% | 37% | 34 | 298.24 | 35 | 307.01 | 36 | 315.78 |
| Lincoln | $62, 061 | 1% | 4% | 10% | 36% | 48% | 34 | 392.93 | 36 | 416.04 | 37 | 427.60 |
| Boston | $33,964 | 14% | 21% | 13% | 30% | 21% | 13061 | 1879.37 | 13417 | 1930.59 | 13609 | 1958.22 |
| Milford | $32,031 | 8% | 27% | 20% | 33% | 12% | 609 | 2078.27 | 617 | 2105.57 | 631 | 2153.34 |
| Framingham | $33,695 | 11% | 23% | 13% | 33% | 20% | 1625 | 2182.20 | 1681 | 2257.40 | 1700 | 2282.91 |
| **State** | **$41,794** | **9.3%** | **23.3%** | **15.3%** | **32%** | **20.10%** | **94220** | **1352.42** | **97964** | **1406.16** | **100158** | **1437.65** |

**Table 12d. Group 7 per-capita incomes, educational attainment, and COVID-19 infection rates between January 1, 2020-July 1, 2020.**

| **Town** | **Per-capita Income** | **Educational Attainment in 2017** | | | | | **COVID-19**  **January 1-June 17, 2020** | | **COVID-19**  **January 1-June 24, 2020** | | **COVID-19**  **January 1-July 1, 2020** | |
| --- | --- | --- | --- | --- | --- | --- | --- | --- | --- | --- | --- | --- |
|  |  | **No HS Dip.** | **HS Dip/**  **GED** | **Some Coll.** | **AS or BA** | **Adv. Deg.** | **Count/ 100k** | **Rate/ 100k** | **Count/ 100k** | **Rate/ 100k** | **Count/ 100k** | **Rate/ 100k** |
| Dover | $115,686 | 2% | 5% | 6% | 40% | 47% | 18 | 345.41 | 18 | 345.41 | 18 | 345.41 |
| Medfield | $67,029 | 1% | 9% | 11% | 42% | 37% | 36 | 315.78 | 36 | 315.78 | 36 | 315.78 |
| Lincoln | $62, 061 | 1% | 4% | 10% | 36% | 48% | 38 | 439.16 | 38 | 439.16 | 40 | 462.27 |
| Boston | $33,964 | 14% | 21% | 13% | 30% | 21% | 13783 | 1983.26 | 13897 | 1999.66 | 13996 | 2013.91 |
| Milford | $32,031 | 8% | 27% | 20% | 33% | 12% | 636 | 2170.41 | 641 | 2187.47 | 646 | 2204.53 |
| Framingham | $33,695 | 11% | 23% | 13% | 33% | 20% | 1707 | 2292.31 | 1720 | 2309.77 | 1734 | 2328.57 |
| **State** | **$41,794** | **9.3%** | **23.3%** | **15.3%** | **32%** | **20.10%** | **101654** | **1459.12** | **102762** | **1475.03** | 103858 | 1490.76 |
