# Supplementary material for: A socioscientific issues approach to ninth-graders’ understanding of COVID-19 on health, wealth, and educational attainments
Source: PLoS One. 2023 Mar 27;18(3):e0280509. doi: 10.1371/journal.pone.0280509 (PMC10045461; doi:10.1371/journal.pone.0280509)
Supplement: S2 File — Fig 3. Group 2. Per-capita income. Fig 4. Group 2. Educational attainment. Fig 5. Group 3. Per-capita income. Fig 6. Group 3. Educational attainment. Fig 7. Group 4. Per-capita income. Fig 8. Group 4. Educational attainment. Fig 9. Group 5. Per-capita income. Fig 10. Group 5. Educational attainment. Fig 11. Group 6. Per-capita income. Fig 12. Group 6. Educational attainment. Fig 13. Group 7. Per-capita income. Fig 14. Group 7. Educational attainment. (DOCX) [file pone.0280509.s016.docx]

S2 File.

**Fig 3. Group 2**. Per-capita income

**Fig 4. Group 2**. Educational attainment

**Fig 5. Group 3.** Per-capita income.

**Fig 6. Group 3.** Educational attainment.

**Fig 7. Group 4.** Per-capita income.

**Fig 8. Group 4.** Educational attainment.

**Fig 9. Group 5.** Per-capita income.

**Fig 10. Group 5.** Educational attainment.

**Fig 11. Group 6**. Per-capita income.

**Fig 12. Group 6.** Educational attainment.

**Fig 13. Group 7.** Per-capita income.

**Fig 14. Group 7.** Educational attainment.
