# Supplementary material for: A socioscientific issues approach to ninth-graders’ understanding of COVID-19 on health, wealth, and educational attainments
Source: PLoS One. 2023 Mar 27;18(3):e0280509. doi: 10.1371/journal.pone.0280509 (PMC10045461; doi:10.1371/journal.pone.0280509)
Supplement: S3 File — (DOCX) [file pone.0280509.s017.docx]

S3 File.

Minimum Data Set.

**What concerns do you have about the CDC facts on Coronavirus?**

1. I have concerns about people I know with preexisting conditions that means they might be more at risk for the disease.
2. Because this disease is so new maybe they don’t have enough facts because we are learning new things about Covid-19 everyday maybe they might put out something that is inaccurate that leads people into thinking something that is far from the truth
3. I am concerned that they do not have the symptoms nor risk groups for Covid-19 on the facts sheet. The facts and instructions are also vague and not specific enough for some to understand or follow.
4. The concerns I have about the CDC facts on Coronavirus is they only tell you how to protect yourself, but they don't discuss what mild and severe symptoms of Coronavirus can range to, which I feel that the public should know about.
5. The main concerns I have about the CDC facts on the coronavirus is that it seems a little outdated. Considering it was created around June, it most likely has changed due to the circumstances right now. However, it does pass the general idea of social distancing and staying at home.

**Do you agree with the suggested ways the CDC suggested to prevent Coronavirus?**

1. I agree with the suggested guidelines to prevent the disease from spreading. There is a lot of scientific evidence supporting the CDC’s ways to prevent Coronavirus.
2. Yes, I agree with the suggested ways the CDC suggested to prevent Coronavirus because if no one takes precautions to prevent the spread then the current circumstances will only get worse over time and if everyone wants to be able to go back to what we once considered as "normal" we all must make sacrifices for the greater good.
3. Mostly, I do agree with the suggested ways the CDC suggested to prevent the Coronavirus. There are some facts that I did not agree with including the one about separating yourself from pets and people in your own home. I think that this something that is difficult to do, and something that I did not know we were supposed to be doing. Some of the ways such as staying at home if you’re sick, and to get medical care if needed, I do agree with. The symptoms for COVID-19 can be bad, and the virus can hurt your respiratory system, so it is important to seek medical attention if needed.

**What if anything concerns you about these suggestions?**

1. I would not be concerned about much except the metal health portion as that can affect most people on this world.
2. Well, a couple of things that would concern me is if there is anything else we could do to prevent the spread of the virus would they take action?
3. I don't like the fact that we have to wear face masks and because of social distancing we can't hug or high five anyone, but I do understand that there are certain sacrifices we have to make whether we like it, which will just help society in the long run, and there's usually light at the end of every tunnel.

**Who is responsible for getting rid of Coronavirus? How do you proposed this should be done?**

1. I think everyone should be responsible for getting rid of Coronavirus. The

disease can only end if everyone does their part. As long as people use the

CDC’s suggestions, the virus should gradually disappear.

1. Society is responsible for getting rid of Coronavirus because the virus spreads among humans, so everyone plays a role in helping to get rid of the virus. The way to do this is by following social distancing guidelines, wearing masks, and limiting in-person contact as much as possible until scientists have figured out some kind of vaccine so that we can all go back to what we used to be able to do and considered to be "normal."
2. I believe that the public are responsible for getting rid of Coronavirus. We can't depend on a vaccine eventually being created, rather we must start following the guidelines that are being put into place. The actions that are being requested aren't, in my opinion, a tasking job. A few simple adjustments that can make all the difference. If everyone were to just wear a mask, stay six feet apart from one another, stay home if they don't feel well, I think that our nation would be in much better standings.

**Answer each question for Graph 1**

**What pattern do you observe?**

1. A pattern I observe is the city's/towns with lower per-capita income, have higher infection rates, and the infection rates increased over time. Another pattern I observe is the city’s/towns with higher per-capita income, have lower infection rates and have either stayed the same or very little increased infection rates.
2. As the per-capita lowers the covid rates raise and with that the cases do too. So, I think ultimately the more privileged places have lower rates.
3. The cities that have the higher income per capita has the lowest rate

**How do you feel about the curves you have observed in the graph?**

1. The curves do not surprise me as most of the richer places are where the curves are smaller and in poorer places it's the opposite.
2. I feel a bit scared because the rate of most of the lines, including Framingham, keep increasing, which means there is a more likely chance to get the virus.
3. I feel surprised but not surprised about the curves I have observed in the graph. I feel this way because seeing how the cities or towns with lower per-capita income have an increase and or higher infection rate than others, doesn’t surprise me because it makes sense. The city’s/towns with low per-capita income have less resources and less money than city’s /towns with higher per-capita income. The city’s/towns with higher per-capita income, have more money and resources to provide for their people, community, and families. Yet, I’m surprised because of how much the infection rates increased over time. In some cities/ towns the infection rates have gotten very high over small amounts of time.

**What does this mean for your attitude towards science?**

1. This means that science should be more mainstream to let all the wage groups of people what risks and other side effects of such a dangerous virus can be.
2. This means that it doesn’t matter how much money you have. Because your city might be in between the poorest.
3. For my attitude towards science, this means that it helps me find that science may be a bit more reliable, and it gives us hope.
4. It helps me see that science helps prove many things and is very reassuring during these times of panic.

**Answer each question for Graph 2**

**What pattern do you observe?**

1. The more educated the city the less the rate of coronavirus in the city
2. If someone is generally less educated than you can see that they are more likely to attain covid-19.
3. Something I observe is even though all of the city/towns have pretty high educational attainment percentages, some towns still have increasing infection rates. Though, those cities/towns with increasing infection rates do have a smaller percentage of education attainment than other cities/towns. A pattern I observe is that all of the cities/downs do increase in infection rates, just some are smaller or larger increases or decreases than others.

**How do you feel about the curves you have observed in the graph?**

1. I feel that we should make it our mission as a society to become more educated as people need to learn more to stay healthy.

**What does this mean for your attitude towards science?**

1. It means for me that science has been doing their job we just need to be able to get more educated in science.
2. For my attitude towards science this means that you learn new things every day and those new things you learn, can affect the way you see your environment and community. It also helps you better understand what’s happening around you and better understand science in general.
3. My attitude is going to improve on wanting to learn more about the science of different things.
4. Science is an efficient way to gather and record data to inform the public of the worsening or improving conditions of life.

**What is your general takeaway from both graphs?**

1. That more privileged places are able to deal with the virus much better than the less fortunate places.
2. My general take away from both graphs is that per-capita income can have a big influence on the a cities or towns resources and help towards pandemic and increasing infection rates and that even if a city or town has a large percentage of educational attainment, they can still have less resources etc., than other cities or towns.
3. I really enjoyed comparing the cases of different cities and what affects the rate of people who get the virus.

**Why are some communities in Massachusetts affected more by COVID-19 than others? Please explain.**

1. Because the cities that have more per-capita income are the most educated in the state, because they have enough money to invest in their education making the people that live there more aware of the virus spreading.
2. Some communities in Massachusetts are affected more by COVID-19 for many reasons. First, some towns’per capita income are somewhat low. This means that their educational attainment will be low as well, which means less kids will learn how to stay safe from the virus. Secondly, most people right now are going through an economic crisis. They aren’t working so they aren’t earning money as much as they need to. This is a problem because they can’t buy the things, they need in order to stay safe such as a mask, cleaning wipes, hand sanitizer, etc. Just look at the amount of people homeless living on the streets right now! They are most likely to catch the virus because they might not have the protection.
3. As we have previously seen on the graphs and the data collected from different cities and towns around Massachusetts, some communities in Massachusetts are affected more by COVID-19 than others due to their per-capita income and percentage at which their population have a BA degree or higher. The lower a city or town’s per-capita income and percentage at which the population has a BA degree or higher, the more that city or town is affected by COVID-19. There can be many interpretations for this pattern, where we can consider that due to the population being less educated, they have to work on the frontline due to not having enough money and being forced to work to bring money and food to their houses and families, and that is what increases the infection by having more people needing to work. There can be also the possibility that due to a higher population not having financial conditions to have a decent healthcare or insurance plan, they are afraid to go to the hospital and treat themselves when they feel bad due to how expensive it is, which leads to a lot of people not taking care of themselves and leading to more infections. Now, for the communities that have a higher per-capita income and education, their population have better conditions to take care of their health and due to them being highly educated, they have enough money to follow the social distancing rules properly without having to worry about money, which becomes proof of a social conflict, where the rich have the best conditions to survive while the poor suffer for their lack of conditions and poverty.
