# Supplementary material for: A socioscientific issues approach to ninth-graders’ understanding of COVID-19 on health, wealth, and educational attainments
Source: PLoS One. 2023 Mar 27;18(3):e0280509. doi: 10.1371/journal.pone.0280509 (PMC10045461; doi:10.1371/journal.pone.0280509)
Supplement: Attachment — Submitted filename: Response to Reviewers.docx [file pone.0280509.s018.docx]

Academic Editor

PLOS ONE

December 22, 2022

Dear Editor,

I am pleased to have been allowed to revise my manuscript entitled "A socioscientific issues approach to ninth-graders' understanding of COVID-19 on health, wealth, and educational attainments" (PONE-D-22-19529). In the revised manuscript, I carefully considered the reviewers' comments and have succinctly made changes in response to the reviewers' comments.

The Center for Excellence in Learning, Teaching, Scholarship, and Service at Framingham State University provided $1,405.00 toward the publication fee of this manuscript. The funder had no role in study design, data collection and analysis, decision to publish, or preparation of the manuscript. The author received no specific funding for this work.

I included a marked-up copy of the manuscript highlighting changes made to the original version. Additionally, I have also uploaded the minimal data set.

Overall, the reviewers' comments were constructive, and I appreciate the feedback on the original manuscript. After addressing the issue raised, the quality of the manuscript has improved.

Below are the actions I have taken to address the concerns raised.

Editor Comments:

1. Please change "female” or "male" to "woman” or "man" as appropriate.

*I changed females and males in the abstract to girls and boys. Please see page 2 of the manuscript.*

2. We note that several of your files are duplicated on your submission. Please remove any unnecessary or old files from your revision, and make sure that only those relevant to the current version of the manuscript are included.

*All duplicated and old files were removed from the submission portal*

3. Thank you for stating the following financial disclosure:

“The funders had no role in study design, data collection and analysis, decision to publish, or preparation of the manuscript”

At this time, please address the following queries:

a. Please clarify the sources of funding (financial or material support) for your study. List the grants or organization that supported your study, including funding received from your institution.

*The Center for Excellence in Learning, Teaching, Scholarship, and Service at Framingham State University provided $1,405.00 toward the publication fee of this manuscript.*

b. State what role the funders took in the study.

*The funder had no role in study design, data collection and analysis, decision to publish, or preparation of the manuscript.*

c. If you did not receive any funding for the study

*The author received no specific funding for this work.*

4. In your Data Availability statement, you have not specified where the minimal data set underlying the results described in your manuscript can be found.

*Please see Appendix for minimal data set.*

Reviewers' comments:

Reviewer's Responses to Questions

Comments to the Author

1. Is the manuscript technically sound, and do the data support the conclusions?

Reviewer #1: Partly

Reviewer #2: Yes

I added to the literature review, changed the research questions, and provided more clarity to the data analysis section of the manuscript. These edits should be enough to shift reviewer # 1 views from partly to yes.

2. Has the statistical analysis been performed appropriately and rigorously?

Reviewer #1: N/A

Reviewer #2: Yes

*As reviewer # 1 indicated, statistical analysis doesn’t apply to this study.*

3. Have the authors made all data underlying the findings in their manuscript fully available?

Reviewer #1: Yes

Reviewer #2: Yes

4. Is the manuscript presented in an intelligible fashion and written in standard English?

Reviewer #1: Yes

Reviewer #2: Yes

5. Review Comments to the Author

Reviewer #1: In my opinion the research questions are out of alignment with the main story of the paper. The research questions seem to be what the students are studying however I think the real story is what the students were thinking about, and their proficiency in making reasoned claims with evidence using the data provided. Also, did this exercise help the students understand the need for using data to inform decision-making? There is a good amount of literature on a justice orientation in science education that can be included as part of the theoretical framework. The author provides a good background and placing the pandemic in context, as well as identifying the need for a study by highlighting the skepticism and questionable claims made by public figures. I would encourage the author to reconsider the main message from the study. In my mind the study should focus on the students’ perspectives and their thoughts rather than determining the rela3onship between the indicators present.

*I revised the research questions and added justice orientation in science to the theoretical framework. See the revised research questions below:*

*Research Question 1.*

*What do middle school students think about when asked to explain the relationships between health in Massachusetts communities and the COVID-19 infection rates?*

*Research Question 2.*

*How proficient are middle school students in using data on COVID-19 infection rates to make evidence-based claims of its relationship to the wealth-health gap among communities in Massachusetts?*

*Research Question 3.*

*Did the students' investigations of COVID-19 infection rates in Massachusetts cities and towns impact their understanding of the need for using data to inform decision-making?*

*Additionally, I have added to the theoretical framework (see pages 5-6 of the manuscript).*

Reviewer #2: - Sampling technique is required along with the actual size of sample.

- this paper is more of qualitative research realm; so in results, discussion the data collected from focused group interview needs to be given more emphasis than document analysis. - the real-life situations and challenges of the sample need to be highlighted more; this would be a better ground to

give suggestive measures in conclusion part. - the paper is nicely drafted.

The research was indeed qualitative. Data were collected primarily through students' written feedback to assigned questions and class discussions on the students’ findings. Though interview data were not used, I expanded the theoretical framework, methods, data generation, data analysis, and the result and discussion section of the manuscript. I believe these edits satisfied the feedback received from reviewer # 2.

Best,

Wardell Powell
